# Primary age‐related tauopathy in a Finnish population‐based study of the oldest old (Vantaa 85+)

**DOI:** 10.1111/nan.12788

**Published:** 2022-01-23

**Authors:** Sara Savola, Karri Kaivola, Anna Raunio, Mia Kero, Mira Mäkelä, Kalle Pärn, Priit Palta, Maarit Tanskanen, Jarno Tuimala, Tuomo Polvikoski, Pentti J. Tienari, Anders Paetau, Liisa Myllykangas

**Affiliations:** ^1^ Department of Pathology University of Helsinki Helsinki Finland; ^2^ Department of Pathology, HUS Diagnostic Center Helsinki University Hospital Helsinki Finland; ^3^ Translational Immunology, Research Programs Unit University of Helsinki Helsinki Finland; ^4^ Department of Neurology University of Helsinki and Helsinki University Hospital Helsinki Finland; ^5^ Institute for Molecular Medicine Finland (FIMM), HiLIFE University of Helsinki Helsinki Finland; ^6^ Translational and Clinical Research Institute Newcastle University Newcastle upon Tyne UK

**Keywords:** amyloid plaques, dementia, neurodegenerative diseases, neurofibrillary tangles, neuropathology, oldest old, PART, population‐based

## Abstract

**Aims:**

Few studies have investigated primary age‐related tauopathy (PART) in a population‐based setting. Here, we assessed its prevalence, genetic background, comorbidities and features of cognitive decline in an unselected elderly population.

**Methods:**

The population‐based Vantaa 85+ study includes all 601 inhabitants of Vantaa aged ≥ 85 years in 1991. Neuropathological assessment was possible in 301. Dementia (DSM IIIR criteria) and Mini‐Mental State Examination (MMSE) scores were assessed at the baseline of the study and follow‐ups. PART subjects were identified according to the criteria by Crary et al and were compared with subjects with mild and severe Alzheimer's disease (AD) neuropathological changes. The effects of other neuropathologies were taken into account using multivariate and sensitivity assays. Genetic analyses included *APOE* genotypes and 29 polymorphisms of the *MAPT* 3′ untranslated region (3*′*UTR region).

**Results:**

The frequency of PART was 20% (*n* = 61/301, definite PART 5%). When PART subjects were compared with those with severe AD pathology, dementia was less common, its age at onset was higher and duration shorter. No such differences were seen when compared with those with milder AD pathology. However, both AD groups showed a steeper decline in MMSE scores in follow‐ups compared with PART. *APOE* ε4 frequency was lower, and *APOE* ε2 frequency higher in the PART group compared with each AD group. The detected nominally significant associations between PART and two *MAPT* 3*′*UTR polymorphisms and haplotypes did not survive Bonferroni correction.

**Conclusions:**

PART is common among very elderly. PART subjects differ from individuals with AD‐type changes in the pattern of cognitive decline, associated genetic and neuropathological features.

## Graphical abstract

Few studies have investigated primary age‐related tauopathy (PART) in a population‐based setting. Here we assessed its prevalence, genetic background, comorbidities, and features of cognitive decline in an unselected elderly population. We found that PART is common among very elderly and differs from Alzheimer's disease in terms of neuropathological features, pattern of cognitive decline, and associated genetic factors and comorbidity pathologies. Thus, it is important to distinguish PART and other novel neuropathological entities from the classical neuropathological diseases.

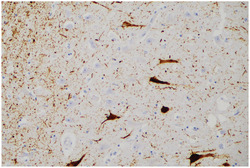

**Key Points**

- Primary age‐related tauopathy (PART) is a common tauopathy in the oldest old.
- PART differs from Alzheimer's disease in terms of neuropathological features, pattern of cognitive decline, and associated genetic factors and comorbidity pathologies.
- It is important to distinguish PART and other novel neuropathological entities from the classical neuropathological diseases in the oldest old.

## INTRODUCTION

The term ‘primary age‐related tauopathy’ (PART) was introduced for the first time in 2014 to describe the common neuropathological finding of Alzheimer's disease (AD)‐type neurofibrillary tangles (NFT) in the medial temporal lobe that, unlike in AD, occur without coexisting amyloid‐β (Aβ) plaques in the brains of the elderly [1]. The existence of this type of ‘hyperphosphorylated tau (HPtau)+/Aβ‐’ pathology has been recognised for a long time, and terms like ‘senile dementia of the neurofibrillary tangle type’ (SD‐NFT) or ‘neurofibrillary tangle‐predominant dementia’ (NFTPD) were previously used to describe this entity [2, 3]. Because it has since been noted that ‘HPtau+/Aβ−’ pathology is also quite common in cognitively unimpaired or mildly impaired elderly [4, 5], the new nomenclature was proposed to include the whole spectrum [1]. In addition to the lack of Aβ plaques, PART also differs neuropathologically from AD in regard to the extent of HPtau pathology [1, 6, 7]. PART is currently classified according to the criteria by Crary et al, as definite PART when Aβ plaques are completely absent (Braak stages I–IV, Thal phase 0), and as possible PART when Aβ plaques are sparse (Braak stages I–IV, Thal phases 1–2), with HPtau pathology in the great majority of cases considered to remain at the level of Braak stage IV or lower (i.e., within the medial temporal lobe) [1]. In contrast, the HPtau pathology in AD is expected to progress further and eventually cover the whole neocortex (Braak stages V–VI) [6, 7, 8].

Because individuals with HPtau+/Aβ− pathology do not meet the neuropathological criteria for AD set by the National Institute on Aging and the Alzheimer's Association (NIA‐AA) guidelines [6, 7], it has been suggested that PART could be a disease entity separate from AD [1, 9, 10]. However, whether or not PART is its own entity or simply an early stage or a subtype of AD has been debated [11, 12]. Arguments that support the existence of PART are differences in the clinical manifestation and genetic factors when compared with AD [9]. Clinically, PART appears to present with either no cognitive impairment or less severe cognitive impairment with slower rates of decline in older individuals when compared with AD [13, 14, 15]. However, assessment of cognitive impairment in PART is complicated by the multiple comorbidities that exist in this age group [1, 16]. In AD genetic research, several genome‐wide association studies (GWAS) during recent years have found dozens of new genetic risk variants tied to AD, with *apolipoprotein E* (*APOE*) still being the most important [17, 18]. Interestingly, PART is not associated with the *APOE* ε4 allele but rather with the *APOE* ε2 allele [19], which is noteworthy because the *APOE* ε4 allele is commonly known as the strongest genetic risk factor for sporadic late‐onset AD [20], whereas *APOE* ε2 might have more of a protective role [21]. It has also been suggested that variation in the *microtubule‐associated protein tau* (*MAPT*) gene is associated with PART [22]. Lastly, a recent study reported that the CA2 region of the hippocampus might be particularly vulnerable in PART [23], suggesting that there might be a difference in the pattern of neurofibrillary degeneration between PART and AD. However, the differences in the pathogenesis that would separate PART from AD are yet to be determined.

There are only a few studies that have assessed PART in a population‐based setting [3, 24, 25]. Therefore, the aim of this study was to carry out a comprehensive analysis of PART in our population‐based study material (the Vantaa 85+ study), in which all the individuals were 85 years or older, thus some of them likely represent so called ‘super agers’. The prevalence of PART has been suggested to be high in this age group [1, 9, 10]. Our focus was on assessing the prevalence, genetic background and features of cognitive decline of PART in an unselected older population and on comparing the findings to individuals with AD‐type neuropathological changes of different severity. Because multiple pathologies often coexist in the brains of the elderly, we also performed multivariate analyses to better understand which neuropathological processes had driven the detected cognitive changes in our study participants, and sensitivity analyses to see if the results changed when subjects with certain brain pathologies or genetic features were excluded.

## MATERIALS AND METHODS

### Participants

The Vantaa 85+ study includes all individuals aged ≥ 85 years, living in the city of Vantaa, Finland, on 1 April 1991. Of the total cohort (*n* = 601), 553 (92%) participated in the baseline study in 1991; 1 could not be contacted, 11 refused to participate and 36 died before examination. Clinical follow‐ups were performed in 1994, 1996 and 1999. The clinical information collected included data about cognitive status, medicated hypertension, medicated type 2 diabetes mellitus, smoking status and blood lipids analysed from nonfasting blood samples using standard laboratory methods [26]. During a 10‐year follow‐up period, 304 autopsies were carried out. This neuropathologically examined subpopulation (*n* = 304) consisted of 252 women and 52 men, and their age at death varied from 85 to 105 years. For the present study, the final neuropathological subsample consisted of 301 individuals (two participants were excluded due to lack of hippocampal samples, and one participant was excluded due to having probable PSP). In this neuropathologically examined subpopulation, DNA samples were available in 279 individuals for *APOE* analysis [27]. Additionally, an analysis of the *MAPT* 3′ untranslated region (3′UTR) and of common *MAPT* haplotypes was performed on 264 participants (see supporting information, Supplementary Methods). Furthermore, to estimate differences in the genetic predisposition for AD in our study participants, we calculated genetic risk scores (GRS) for AD representing the burden of AD genetic risk variants (see supporting information, Supplementary Methods).

### Assessment of cognitive impairment

Assessment of cognitive impairment during the clinical examinations has been described previously [28]. Dementia was diagnosed using the DSM IIIR criteria, provided that the duration of dementia had been at least 3 months prior to the examination. Cognitive function was also assessed using the Mini‐Mental State Examination (MMSE) scores at the baseline study (in 1991, *n* = 283) and the MMSE scores of still living participants at the follow‐ups in 1994 (*n* = 134), 1996 (*n* = 82) and 1999 (*n* = 24) [29].

### Neuropathological procedures and assessment

For the present study, we determined Braak HPtau stages and Thal Aβ phases using immunohistochemistry (Table S1). This evaluation followed the NIA‐AA guidelines for the neuropathological assessment of AD [6, 7]. Samples used for the assessment of HPtau pathology included the hippocampus at the level of the lateral geniculate body, middle temporal gyrus and occipital cortex at Brodmann areas 17 and 18. The hippocampal sections have been previously stained immunohistochemically with HPtau (AT8) [30]. Here, 4‐μm sections of the temporal and occipital tissue blocks were stained immunohistochemically with phospho‐tau (Ser202, Thr205) mouse monoclonal antibody (clone AT8, 1:1000, Invitrogen/Thermo Fischer Scientific, Waltham, Massachusetts, USA) using the immunostainer LabVision and Dako EnVision™ FLEX detection system with EnVision™ FLEX+ Mouse Linker. Epitope retrieval was done with BioCare Medical Decloaking Chamber™ NxGen (95°C) using TE‐buffer (pH 9). The Braak HPtau stages (0–VI) were then determined microscopically using the modified Braak staging scheme [31, 32]. Assessment of the extent of Aβ pathology according to the Thal phases [33] was performed using samples from the middle frontal gyrus, hippocampus at the level of the lateral geniculate body, basal ganglia at the level of the basal nucleus of Meynert, mesencephalon at the level of the superior colliculus and right cerebellar hemisphere including the dentate nucleus. Immunohistochemical staining of the frontal, hippocampal and cerebellar samples with an Aβ antibody has been described previously [34], and the basal ganglia and mesencephalon were now stained using the same method. Additionally, samples that had been Aβ‐negative or scantly positive in the original study [34] were restained with a more sensitive detection kit. The 4‐μm‐thick sections were stained immunohistochemically with anti‐β‐amyloid mouse monoclonal antibody (clone 4G8, residues 17–24, 1:14,000, BioLegend, San Diego, CA, USA) using the immunostainer LabVision and Dako EnVision™ FLEX detection system. Epitope retrieval was done with a BioCare Medical Decloaking Chamber™ NxGen (95°C, 20 min) using TE‐buffer (pH 9), followed by 99% formic acid treatment (5 min). All samples were then microscopically determined as either positive, scantily positive or negative for Aβ plaques, according to the method described by Thal et al. [33]

Assessment of ‘Consortium to Establish a Registry for AD’ (CERAD) neuritic plaque score [35], types and severity of cerebral amyloid angiopathy (CAA) [34, 36], Lewy‐related pathology (LRP) [37], ‘limbic predominant age‐related TDP‐43 encephalopathy neuropathological change with hippocampal sclerosis’ (LATE‐NC with HS) [30] and cerebral infarcts [26] have been described previously. The screening protocol for argyrophilic grains is described in detail in the supporting information, Supplementary Methods.

For statistical comparison, participants were divided into three groups: (I) a combined PART group, consisting of both definite PART and possible PART individuals (Braak I–IV, Thal 0–2); (II) a low AD group, consisting of individuals with mild AD‐type neuropathological changes (Braak I–IV, Thal 3–5); and (III) a high AD group, consisting of individuals with severe AD‐type neuropathological changes (Braak V–VI, Thal 3–5). PART was classified according to criteria suggested by Crary et al [1]. Because of the small number of definite PART subjects, the definite and possible PART subjects were analysed together to retain statistical power, even though it was noted when one or the other may have been driving the statistical significance of the results. Four participants did not meet the criteria for any of the groups and were thus excluded from analyses, that is, one had no HPtau pathology (Braak 0), and three had Thal phases 0–2, but Braak stages V–VI.

### Statistical analyses

The statistical analyses were performed using IBM SPSS Statistics version 26. We used the Chi^2^‐test to assess if there were any differences in various categorical variables (sex, dementia status, smoking, medication for hypertension, medication for type 2 diabetes mellitus, *APOE* genotype, Tau haplotype, LRP, CAA, LATE‐NC with HS, argyrophilic grain disease [AGD] and cerebral infarcts) between PART and the AD (low and high) groups. If the expected count was less than five, Fisher's exact test was used. Mann–Whitney *U* test was used to compare medians of continuous variables between the groups (age at death, age at onset of dementia, duration of dementia, baseline MMSE and cholesterol). We then used regression analysis to adjust the results for age and sex. Additionally, we compared changes in MMSE scores over time in the different groups. Thus, we used linear regression analysis with PART vs low/high AD, age and sex as covariates to compare the change in MMSE scores between the baseline study in 1991 and follow‐ups in 1994, 1996 and 1999, respectively. Next, to explore which other neuropathological factors might have been driving the cognitive changes in our study subjects, we performed multivariate regression analyses (with dementia status or MMSE scores as the dependent variable) by adding comorbidity pathologies (LATE‐NC with HS, AGD, diffuse neocortical and limbic predominant LRP, and small cortical infarcts) as additional covariates to our age‐ and sex‐adjusted models. Small cortical infarcts were chosen over other cerebral infarcts because they showed the strongest association with dementia [36]. We also performed sensitivity analyses by excluding certain subjects based on their comorbidity pathologies or genetic features from the age‐ and sex‐adjusted regression analyses to see if this made any difference to the results. Statistical analyses of the *MAPT* 3′UTR gene region are described in the supporting information (see Supplementary Methods).

## RESULTS

### Demographics

Table 1 shows the demographic information of our study participants (*n* = 301). Altogether, 61 (20%) participants were classified as PART (16 (5%) as definite PART, and 45 (15%) as possible PART), whereas 133 (44%) participants met the criteria for the low AD group, and 103 (34%) for the high AD group. Females predominated (83% vs 17%) in this very elderly population‐based cohort, but there were no gender‐related significant differences between the groups. There was also no association between the groups and age at death, smoking status, cholesterol values or use of medication for hypertension, but PART subjects were slightly more often medicated for type 2 diabetes than the high AD subjects (0.01 < *p* < 0.05, Table 1).

**TABLE 1:** Clinical characteristics of all study participants, and of PART subjects, subjects with low AD‐type neuropathological changes and subjects with high AD‐type neuropathological changes

Variable	All participants	All PART	Definite PART	Possible PART	Low AD	High AD	*p* all PART vs low AD	*p* all PART vs high AD
Number of subjects, *n* (%)	301^a^	61 (20.3)	16 (5.3)	45 (15.0)	133 (44.2)	103 (34.2)		
Sex								
Female/male, *n* (%)	250 (83.1)/51 (16.9)	51 (83.6)/10 (16.4)	13 (81.3)/3 (18.8)	38 (84.4)/7 (15.6)	110 (82.7)/23 (17.3)	85 (82.5)/18 (17.5)	NS	NS
Age at death								
Median (IQR)	92.1 (5.2)	91.7 (5.6)	91.0 (2.8)	93.0 (5.9)	92.7 (5.1)	91.4 (5.0)	NS	NS
Dementia^b^								
Yes, *n* (%)	195 (64.8)	29 (47.5)	8 (50.0)	21 (46.7)	73 (54.9)	90 (87.4)	NS	*p* < 0.001
Age at onset of dementia								
Median (IQR)	87.4 (5.4)	88.6 (7.0)	86.2 (4.0)	89.4 (6.3)	88.5 (5.6)	86.5 (5.5)	NS	0.001 ≤ *p* < 0.01
Duration of dementia								
Median (IQR)	4.3 (5.2)	3.3 (2.9)	4.8 (2.4)	2.8 (2.4)	4.1 (4.9)	4.9 (6.1)	NS	0.001 ≤ *p* < 0.01
MMSE^c^ median (IQR)								
Baseline 1991	18 (14)	21 (12)	17 (13)	21 (11.75)	21 (12)	13 (21)	NS	*p* < 0.001
Change in MMSE^d^ median (IQR)								
1991 vs follow‐up 1994	−3 (6)	−1 (5)	−1 (2)	−1.5 (6)	−3 (5)	−3.5 (7.3)	0.01 < *p* < 0.05	0.001 ≤ *p* < 0.01
1991 vs follow‐up 1996	−6 (9)	−2 (6)	−2 (0)	−1 (7)	−6 (10)	−9 (7.5)	0.01 < *p* < 0.05	0.001 ≤ *p* < 0.01
1991 vs follow‐up 1999	−6 (9)	−4 (11.3)	−1 (*n* = 1)	−7 (*n* = 3)	−5 (8)	−10 (8)	NA	NA
Smokers^e^								
Yes, *n* (%)	37 (14.7)	9 (16.1)	3 (21.4)	6 (14.3)	18 (15.8)	10 (12.8)	NS	NS
Cholesterol^f^ median (IQR)								
HDL (mmol/L)	0.96 (0.36)	0.99 (0.33)	1.01 (0.31)	0.99 (0.35)	0.97 (0.42)	0.93 (0.36)	NS	NS
LDL (mmol/L)	3.48 (1.47)	3.24 (1.69)	3.09 (2.19)	3.26 (1.66)	3.46 (1.45)	3.57 (1.35)	NS	NS
TGL (mmol/L)	1.63 (1.08)	1.83 (0.80)	2.15 (1.01)	1.68 (0.70)	1.57 (1.24)	1.59 (0.99)	NS	NS
Hypertension^g^								
Yes, *n* (%)	77 (25.7)	17 (27.9)	3 (18.8)	14 (31.1)	37 (27.8)	23 (22.5)	NS	NS
DM2^h^								
Yes, *n* (%)	64 (21.3)	18 (29.5)	6 (37.5)	12 (26.7)	28 (21.1)	17 (16.5)	NS	0.01 < *p* < 0.05

*Note*: Results of association analyses with different variables, adjusted for age and sex.

Abbreviations: AD, Alzheimer's disease; DM2, type 2 diabetes mellitus; HDL, high‐density lipoprotein; IQR, interquartile range; LDL, low‐density lipoprotein; MMSE, Mini‐Mental State Examination; NA, not applicable; NS, no statistical significance; PART, primary age‐related tauopathy; TGL, triglycerides.

^a^ Four participants did not meet criteria for the PART, low AD or high AD groups.

^b^ Frequency of dementia was also calculated by excluding other comorbidities (see Table S10).

^c^ Median MMSE scores at baseline (1991) for each group out of a maximum of 30 points. MMSE scores were available at baseline in 283 participants.

^d^ Change in MMSE score between two points in time: baseline (1991) vs each follow‐up (1994, 1996 and 1999). MMSE scores were available at follow‐ups in 134 (1994), 82 (1996) and 24 (1999) participants.

^e^ Smoker status known in 252/301 participants.

^f^ Cholesterol values available in 263/301 participants at baseline.

^g^ Participants using blood pressure medication, data available in 300/301 participants.

^h^ Participants using medication for type 2 diabetes.

### Comorbid pathologies

Neuropathological features of our study participants are shown in Table 2. Of the 301 participants, all but one (99.7%) had some degree of HPtau pathology in the examined brain regions, whereas about 20% had no or only sparse Aβ deposits. Only two (13%) definite PART subjects had any LRP, compared with 38% of possible PART subjects, 40% of low AD subjects and 49% of high AD subjects. Diffuse neocortical LRP was significantly more common in the high AD group compared with the combined PART group (22% vs 5%, 0.001 ≤ *p* < 0.01), and comparison of low AD and PART showed a similar trend (13% vs 5% 0.05 ≤ *p* < 0.1, Table 2) but was not statistically significant. No amygdala‐predominant LRP subjects were found within the PART group, whereas most (7/10) amygdala‐predominant LRP subjects were in the high AD group. When comparing the type of CAA pathology between the groups, we found that the majority of the PART subjects did not have any CAA at all (94% of definite PART subjects and 62% of possible PART subjects), and only one PART subject (i.e., in the possible PART group) had CAA‐Type 1 (CAA with capillary Aβ), whereas the rest had CAA‐Type 2 (CAA without capillary Aβ) (Table 2). In contrast, the majority of individuals in the high AD (93%) and low AD (80%) groups had CAA. The high AD group had the highest percentage of CAA‐Type 1 subjects (49%). LATE‐NC with HS was somewhat more common in the high AD group compared with the combined PART group (21% vs 8%, 0.01 < *p* < 0.05), and none of the definite PART subjects had LATE‐NC with HS. AGD, on the other hand, was more common in the combined PART group compared with high AD (34% vs 18%, 0.01 < *p* < 0.05). The frequency of cerebral infarcts did not differ significantly between the groups.

**TABLE 2:** Neuropathological features of all study participants, and of PART subjects, subjects with low AD‐type neuropathological changes and subjects with high AD‐type neuropathological changes

Variable	All participants	All PART	Definite PART	Possible PART	Low AD	High AD	*p* all PART vs low AD	*p* all PART vs high AD
CERAD score^a^, *n* (%)							*p* < 0.001	*p* < 0.001
None	69 (22.9)	55 (90.2)	16 (100.0)	39 (86.7)	10 (7.5)	1 (1.0)		
Sparse	33 (11.0)	5 (8.2)	0 (0.0)	5 (11.1)	27 (20.1)	1 (1.0)		
Moderate	160 (53.2)	1 (1.6)	0 (0.0)	1 (2.2)	89 (66.4)	70 (68.0)		
Frequent	39 (13.0)	0 (0.0)	0 (0.0)	0 (0.0)	8 (6.0)	31 (30.0)		
Braak NFT stage^b^, *n* (%)							0.001 ≤ *p* < 0.01	NA
0–II	53 (17.6)	20 (32.8)	7 (43.8)	13 (28.9)	32 (24.1)	0 (0.0)		
III–IV	142 (47.2)	41 (67.2)	9 (56.2)	32 (71.1)	101 (75.9)	0 (0.0)		
V–VI	106 (35.2)	0 (0.0)	0 (0.0)	0 (0.0)	0 (0.0)	103 (100.0)		
Thal phase^c^, *n* (%)							NA	NA
0	17 (5.6)	16 (26.2)	16 (100.0)	0 (0.0)	0 (0.0)	0 (0.0)		
1–2	47 (15.6)	45 (73.8)	0 (0.0)	45 (100.0)	0 (0.0)	0 (0.0)		
3	27 (9.0)	0 (0.0)	0 (0.0)	0 (0.0)	26 (19.5)	3 (2.9)		
4–5	210 (69.8)	0 (0.0)	0 (0.0)	0 (0.0)	107 (80.5)	100 (97.1)		
LRP^d^, *n* (%)								
None	178 (59.1)	42 (68.9)	14 (87.5)	28 (62.2)	80 (60.2)	53 (51.5)		
Brainstem predominant	19 (6.3)	6 (9.8)	1 (6.3)	5 (11.1)	10 (7.5)	3 (2.9)	NS	NS
Limbic predominant	40 (13.3)	9 (14.8)	1 (6.3)	8 (17.8)	15 (11.3)	15 (14.6)	NS	NS
Diffuse neocortical	43 (14.3)	3 (4.9)	0 (0.0)	3 (6.7)	17 (12.8)	23 (22.3)	0.05 ≤ *p* < 0.1	0.001 ≤ *p* < 0.01
Amygdala predominant	10 (3.3)	0 (0.0)	0 (0.0)	0 (0.0)	3 (2.3)	7 (6.8)	NA	NA
Non classifiable	11 (3.7)	1 (1.6)	0 (0.0)	1 (2.2)	8 (6.0)	2 (1.9)	NS	NS
CAA^e^, *n* (%)								
CAA‐Type 1	85 (28.6)	1 (1.6)	0 (0.0)	1 (2.2)	34 (25.6)	50 (48.5)	0.001 ≤ *p* < 0.01	p < 0.001
CAA‐Type 2	135 (45.5)	17 (27.9)	1 (6.2)	16 (35.6)	69 (51.9)	46 (44.7)	0.001 ≤ *p* < 0.01	0.01 < p < 0.05
No CAA	77 (25.9)	43 (70.5)	15 (93.8)	28 (62.2)	26 (19.5)	7 (6.8)	p < 0.001	p < 0.001
LATE‐NC with HS^f^, *n* (%)	47(15.6)	5 (8.2)	0 (0.0)	5 (11.1)	20 (15.0)	22 (21.4)	NS	0.01 < p < 0.05
AGD^g^, *n* (%)	81 (27.0)	21 (34.4)	4 (25.0)	17 (37.8)	41 (31.1)	18 (17.5)	NS	0.01 < p < 0.05
Cerebral infarct^h^, *n* (%)								
All regions	162 (53.8)	34 (55.7)	8 (50.0)	26 (57.8)	76 (57.6)	49 (47.6)	NS	NS
Small cortical	57 (18.9)	8 (13.1)	2 (12.5)	6 (13.3)	28 (21.1)	20 (19.4)	NS	NS
Large cortical	51 (16.9)	13 (21.3)	3 (18.8)	9 (0.2)	25 (18.8)	14 (13.6)	NS	NS
Small white matter	44 (14.6)	11 (18.0)	1 (6.3)	10 (22.2)	21 (15.8)	12 (11.7)	NS	NS
Large white matter	6 (2.0)	3 (4.9)	1 (6.3)	2 (4.4)	3 (2.3)	0 (0.0)	NS	NS
Small basal ganglia	60 (19.9)	11 (18.0)	2 (12.5)	9 (0.20)	30 (22.6)	18 (17.5)	NS	NS
Large basal ganglia	1 (0.3)	0 (0.0)	0 (0.0)	0 (0.0)	1 (0.8)	0 (0.0)	NS	NS
Small brain stem	13 (4.3)	2 (3.3)	1 (6.3)	1 (2.2)	8 (6.0)	3 (2.9)	NS	NS
Large brain stem	1 (0.3)	0 (0.0)	0 (0.0)	0 (0.0)	1 (0.8)	0 (0.0)	NS	NS
Small cerebellum	53 (17.6)	12 (19.7)	2 (12.5)	10 (22.2)	23 (17.3)	17 (16.5)	NS	NS
Large cerebellum	15 (5.0)	2 (3.3)	1 (6.3)	1 (2.2)	7 (5.3)	6 (5.8)	NS	NS
Anterior circulation	121(40.2)	27 (44.3)	6 (37.5)	21 (46.7)	56 (42.1)	36 (35.0)	NS	NS
Posterior circulation	98 (32.6)	21 (34.4)	6 (37.5)	15 (33.3)	48 (36.1)	28 (27.2)	NS	NS

*Note*: Results of association analyses with different variables, adjusted for age and sex.

Abbreviations: AD, Alzheimer's disease; AGD, argyrophilic grain disease; CAA, cerebral amyloid angiopathy; CERAD, Consortium to Establish a Registry for AD; LATE‐NC with HS, limbic predominant age‐related TDP‐43 encephalopathy neuropathological change with hippocampal sclerosis; LRP, Lewy‐related pathology; NA, not applicable; NFT, neurofibrillary tangle; NS, no statistical significance; PART, primary age‐related tauopathy.

^a^ Neuropathological protocol for scoring neuritic plaques, data published previously [35].

^b^ Modified Braak staging scheme for HPtau pathology [31, 32].

^c^ Staging scheme for phases of Aβ‐depositions by Thal et al. [33]

^d^ Lewy‐related pathology. DLB Consortium classification for LRP, data published previously [37]. Statistical analysis was performed by comparing the other groups to the ‘no LRP’ group.

^e^ Cerebral amyloid angiopathy, data published previously [34]. Data available in 297/301 participants.

^f^ Limbic‐predominant age‐related TDP‐43 encephalopathy neuropathological change with hippocampal sclerosis. Data published previously [30].

^g^ Argyrophilic grain disease. *n* = 276.

^h^ Data available in 258/301 participants. Data published previously [26].

### Cognitive decline

Overall, 195/301 (65%) of the participants developed dementia (Table 1). Subjects in the combined PART group were less likely to have dementia (48% vs 87%, *p*  <  0.001), and the onset of dementia occurred later in life (mean age 88.6 vs 86.5, 0.001 ≤ *p* < 0.01) and had a shorter duration (mean duration in years 3.3 vs 4.9, 0.001 ≤ *p* < 0.01) compared with high AD subjects. As seen in Table 1, the difference in disease onset and duration derived mainly from the possible PART group. The frequency of dementia, age at onset and duration of dementia did not differ significantly between the combined PART and low AD groups (Table 1).

MMSE scores at the baseline study (in 1991) were higher in the combined PART group compared with the high AD group (median 21 vs 13, *p* < 0.001), but there was no difference in baseline MMSE scores between the combined PART and low AD group (Tables 1 and S2). However, in comparisons between the baseline MMSE scores and the follow‐up MMSE scores in still living participants (Tables 1 and S2), we observed that the MMSE scores declined more slowly in PART subjects compared with both the high AD (*B* = −3.59, 95% CI = −5.83 to −1.36, *p* = 0.002) and low AD (*B* = −2.69, 95% CI = −4.76 to −0.61, *p* = 0.012) subjects between 1991 and 1994 (*n* = 134), and also between 1991 and 1996 (*n* = 82) (PART vs high AD: *B* = −5.33, 95% CI = −9.10 to −1.55, *p* = 0.006. PART vs low AD: *B* = −4.00, 95% CI = −7.52 to −0.48, *p* = 0.026). This finding is visualised in Figure S1. When comparing baseline MMSE scores with follow‐up MMSE scores in 1999, the sample size (24 surviving study participants) was too small at this point to perform a reliable analysis.

Results of the multivariate regression analyses are shown in Table S2. After controlling our main age‐ and sex‐adjusted results for additional brain pathologies, we found that the high AD subjects remained more likely to develop dementia and have lower MMSE scores at baseline than the PART subjects. Additionally, LATE‐NC with HS, diffuse neocortical and limbic predominant LRP and small cortical infarcts also correlated with cognitive decline (dementia and/or lower MMSE score at baseline) in our study subjects (Table S2). The finding that MMSE scores declined more slowly in PART subjects compared with low and high AD subjects between 1991 and 1994 also did not change in the multi‐adjusted model, but when comparing MMSE scores in 1991 and 1996, statistical significance was no longer present.

Results of the sensitivity analyses are seen in Table S3. Excluding comorbid pathologies that were likely to affect cognitive decline mostly did not affect our main age‐ and sex‐adjusted models assessing cognitive impairment. The only change was excluding subjects with diffuse neocortical and limbic predominant LRP, LATE‐NC with HS, small cortical infarcts and severe CAA annulled the statistically significant difference between PART and low AD when comparing MMSE scores in 1991 and 1996, as predicted by the multi‐adjusted model, but the statistical significance in comparison between PART and high AD remained.

### *APOE* genotypes and variation in *MAPT*

The *APOE* ε4 allele was significantly more frequent in both the high AD group (in 54% vs 5% of the subjects, *p* < 0.001) and low AD group (in 28% vs 5% of the subjects, 0.001 ≤ *p* < 0.01) compared with the PART group (Table 3). None of the definite PART subjects had an *APOE* ε4 allele. The *APOE* ε2 allele on the other hand was more frequent in the PART group compared with the high AD group (in 24% vs 6% of the subjects, 0.001 ≤ *p* < 0.01) and to the low AD group (in 24% vs 12% of the subjects, 0.01 < *p* < 0.05). In sensitivity analyses, when excluding all subjects with *APOE* ε2 and ε4 from our main age‐ and sex‐adjusted models assessing cognitive impairment (leaving subjects with *APOE* ε3ε3), all the statistically significant results were either weakened or annulled (Table S3). Results of the AD GRS calculations are described in supporting information (Supplementary Results) and visualised in Figures S2–S3. Excluding PART group AD GRS outliers in the sensitivity analysis made no difference in the outcome of the main analysis (Table S3). In the analysis of ancestral tau haplotypes, there was no difference in *Tau H1/H2* frequencies between the groups (Table 3). Nominally significant associations were found with two *MAPT* 3′UTR region polymorphisms (rs7521 and rs564954259) when PART and low AD were compared (Table S4–S5), but these findings did not survive Bonferroni correction. For descriptive purposes, we performed haplotype analysis of the *MAPT* 3′UTR region, which identified two nominally significant haplotypes when comparing PART and low AD and one nominally significant haplotype when comparing PART and high AD. These results did not survive Bonferroni correction (Tables S6–S7).

**TABLE 3:** *APOE* genotypes, allele ε4 and ε2 frequencies, and ancestral tau haplotypes (H1/H2) of study participants with genetic data

Variable	All participants, *n* = 279	All PART, *n* = 59	Definite PART, *n* = 15	Possible PART, *n* = 44	Low AD, *n* = 120	High AD, *n* = 96	*p* all PART vs low AD	*p* all PART vs high AD
*APOE* genotype^a^, *n* (%)							*p <* 0.001	*p <* 0.001
2/2	1 (0.4)	1 (1.7)	1 (6.7)	0 (0.0)	0 (0.0)	0 (0.0)		
2/3	28 (10.0)	12 (20.3)	2 (13.3)	10 (22.7)	11 (9.2)	4 (4.2)		
3/3	162 (58.1)	43 (72.9)	12 (80.0)	31 (70.5)	76 (63.3)	40 (41.7)		
2/4	6 (2.2)	1 (1.7)	0 (0.0)	1 (2.3)	3 (2.5)	2 (2.1)		
3/4	80 (28.7)	2 (3.4)	0 (0.0)	2 (4.5)	30 (25.0)	48 (50.0)		
4/4	2 (0.7)	0 (0.0)	0 (0.0)	0 (0.0)	0 (0.0)	2 (2.1)		
*APOE* ε4 allele^b^								
Yes, *n* (%)	88 (31.5)	3 (5.1)	0 (0.0)	3 (6.8)	33 (27.5)	52 (54.2)	0.001 ≤ *p* < 0.01	*p* < 0.001
*APOE* ε2 allele^b^								
Yes, *n* (%)	35 (12.5)	14 (23.7)	3 (20.0)	11 (25.0)	14 (11.7)	6 (6.3)	0.01 < *p* < 0.05	0.001 ≤ *p* < 0.01
Tau haplotype^c^, *n* (%)								
H1/H1	229 (84.2)	47 (82.5)	13 (92.9)	34 (79.1)	94 (81.0)	85 (89.5)	NS	NS
H1/H2	39 (14.3)	9 (15.8)	1 (7.1)	8 (18.6)	19 (16.4)	10 (10.5)	NS	NS
H2/H2	4 (1.5)	1 (1.8)	0 (0.0)	1 (2.3)	3 (2.6)	0 (0.0)	NS	NS

Abbreviations: AD, Alzheimer's disease; NS, no statistical significance; PART, primary age‐related tauopathy.

^a^ Statistical analysis was performed using Fisher's exact test.

^b^ Statistical analysis was performed using binary logistic regression (adjusted for age and sex).

^c^ Tau haplotype data were available in 272/301 participants. Statistical analysis was performed using binary logistic regression (adjusted for age and sex).

## DISCUSSION

Our population‐based study supports the previous notions that basically all brains show some degree of HPtau pathology at old age, whereas a substantial proportion lack Aβ deposits [9, 24, 25]. Thus, HPtau accumulation in the absence of Aβ pathology, that is, PART, appears common. Accordingly, we found that the prevalence of PART (definite and possible) in the Vantaa 85+ study cohort was 20%, of which definite PART represented about a quarter (i.e., a prevalence of 5%). Reports of the frequency of HPtau+/Aβ− pathology in elderly in previous studies have varied, ranging from a few percent to about 20%, and even up to 30%–40% [1, 2, 3, 4, 5, 9, 10, 13, 24, 25, 38, 39, 40]. Most reports of the 80+ age group have shown a prevalence of ~20%. However, comparing the prevalence of PART between different studies is not straightforward. First, most studies reporting HPtau+/Aβ− frequencies have not been population‐ or community‐based. Second, there has been some variability between different studies regarding neuropathological inclusion/exclusion criteria, sample size, age, ethnicity and methodology for assessing cognitive decline, which may explain some of the variation in reports of prevalence. For example, in the Japanese population‐based Hisayama study [3], the prevalence of SD‐NFT was only 3.9% among individuals with dementia. However, all these individuals had dementia and a Braak stage of IV, thus representing individuals on the more severe end of the HPtau+/Aβ− spectrum. In cognitively normal elderly alone, however, HPtau+/Aβ− pathology has been shown to be quite common [4], implying that the whole spectrum can be much wider. This was seen in a report of a neuropathologically examined subpopulation (*n* = 233) of the community‐based VITA study, in which approximately 31% of the >75‐year‐old participants were HPtau+/Aβ− subjects [24]. Similarly, in the population‐based 90+ study, which also used a small neuropathological subpopulation (*n* = 185) of a larger population, the frequency of definite PART was recently reported as 18%, and the frequency of possible PART was 23% [25]. Interestingly, the frequency of definite PART was much higher in the 90+ study when compared with the present study (5%). In the 90+ study, the mean age at death (97.7 vs 92.1 years) and the proportion of highly educated were higher (47% vs <10%) when compared with the Vantaa 85+ study, which might explain the discrepancy in definite PART frequencies.

Multiple simultaneous brain pathologies are common in very elderly populations [41, 42]. We were able to pinpoint some differences in the frequency of certain comorbid pathologies between PART and AD, especially when looking at definite PART vs high AD subjects. First, our PART subjects were less likely to have LRP than AD subjects, which is in line with what has been demonstrated by some previous studies [13, 43]. This finding is expected because LRP often exists in combination with AD, and it has even been hypothesised that there exists a biologically distinct AD‐associated LRP type with an amygdala‐based progression pattern [37, 44, 45]. In this context, it was interesting to find that most of our amygdala‐predominant LRP subjects were in the high AD group (7/10), whereas none of them were in the PART group. Similarly, it has also been demonstrated that CAA, especially CAA‐Type 1, is less prevalent in PART when compared with AD [2, 3, 19], which was also the case in our study. This fits the fact that CAA‐Type 1 is known to associate with the *APOE* ε4 allele [46]. Furthermore, in a study by Josephs et al [43], it was reported that hippocampal sclerosis was quite rare in PART subjects (6%), whereas AGD was quite common (about 30%), also consistent with our findings. The association between PART and AGD is noteworthy. AGD is a poorly understood four‐repeat (4R) tauopathy occurring in old age and is known to associate strongly with the *APOE* ε2 allele [47, 48], and we therefore postulate that the linkage between PART and AGD in our study could be mediated by the *APOE* ε2 allele. Another commonality between PART and AGD is that they both have been shown to exhibit a selective vulnerability of the CA2 region of the hippocampus, but because PART is both a 3R and 4R tauopathy rather than just a 4R tauopathy, these two most probably represent two different disease processes [23, 48]. The rarity of LATE‐NC with HS in PART is in turn a clear difference from AD, because AD‐type neuropathological changes and LATE‐NC with HS are often comorbidities [49], as demonstrated by our study (>20% of high AD subjects had LATE‐NC with HS). The frequency of cerebrovascular lesions on the other hand did not differ between our PART and AD subjects. This has also been the case in some previous studies [2, 13], even though another previous study showed that PART subjects had a higher frequency of vascular brain injury compared with AD [15]. Taken together, the differences in the burden of comorbidity pathologies between PART and AD that we and others have observed could indicate that these entities might be formed by two different mechanisms, although some of these findings could reflect an overlap in the diagnostic criteria among the classics and novelties of neurodegenerative diseases in this still evolving field of research.

Many previous studies have found that PART and AD show clear differences in cognitive abilities, with PART subjects more often having either no dementia or a more slowly progressing or milder cognitive impairment in older individuals compared with AD [2, 5, 13, 14, 15, 50, 51]. When comparing PART and high AD, we were able to replicate these findings, but when comparing PART and low AD, the differences mostly disappeared. Because severity of cognitive impairment in AD correlates best with the number of isocortical NFTs [52, 53], it is not surprising that the difference in the frequency of dementia between HPtau+/Aβ− and ‘HPtau+/Aβ+’ subjects disappeared when comparisons were made without neocortical Braak stages (V–VI). Additionally, recent studies have found that the Braak HPtau staging scheme, which focuses on the regional distribution of HPtau pathology rather than quantification, does not correlate with cognitive decline in PART subjects as well as it does in AD subjects [16, 23]. In the study by Iida et al [16], it was even shown that computer‐derived quantitative assessment of HPtau burden was a better predictor of cognitive impairment than the Braak stages in PART subjects. Because we used the Braak HPtau staging scheme to evaluate disease severity in PART, this might be one explanation why we were not able to find significant differences in cognitive status between PART and low AD. Still, like the high AD subjects, even the low AD subjects showed a steeper decline in MMSE scores during follow‐up than PART subjects in our main age‐ and sex‐adjusted analyses, indicating a difference in disease progression between PART and AD.

Evaluating the effect that PART pathology has on cognitive decline is complicated by the high frequency of comorbid brain pathologies seen in this age group [1]. In our multivariate analyses, we found that in addition to severe AD pathology correlating significantly with cognitive impairment, some other brain pathologies (i.e., LATE‐NC with HS, diffuse neocortical and limbic predominant LRP and small cortical infarcts) also correlated independently with cognitive impairment in our study subjects. Additionally, when taking into account these other brain pathologies, the statistically significant difference in the decline of MMSE scores between 1991 and 1996 disappeared in comparisons between PART and both low and high AD, although this might reflect a too small sample size in follow‐up for reliable multiple regression analysis (*n* = 82 in 1996). In previous studies, it has been shown that coexistence of other comorbidities such as cerebrovascular disease associates with cognitive decline in PART subjects [16, 51]. Still, it appears that some subjects with HPtau+/Aβ− pathology on the more severe end of the PART spectrum can develop significant dementia in the absence of other explanatory features [1, 3, 51]. To examine this, we recalculated the percentages of dementia in our study subjects after exclusion of some burdensome comorbidities and found that the frequency of dementia among our PART subjects lessened somewhat (Table S10). Even then, a substantial portion of our PART subjects (36%) remained with dementia. Therefore, in light of the current and previous studies, we conclude that even though the role of PART in dementia seems to be smaller in comparison with other more burdensome brain pathologies, it still appears be able to independently cause noteworthy cognitive impairment.

In accordance with findings of the present study, PART has previously shown the reverse association with *APOE* genotypes compared with AD, with *APOE* ε4 being less common and *APOE* ε2 being more common in PART [13, 19, 54, 55, 56]. It is of note that the *APOE* ε2/ε3 genotype has been associated with the lowest Aβ deposition, which is in line with the definition of PART [57]. The difference in this key genetic factor in PART vs classical AD indicates that there might be differences in Aβ metabolism between these disease entities and that PART might thus be a separate tauopathy from AD. As for the variation in the *MAPT* gene previously associated with PART, the study by Santa‐Maria et al [22] suggested that TPD had a strong protective association with the *MAPT H2* haplotype when compared with ‘successful cerebral ageing’. Additionally, the study found an association between TPD and two polymorphisms in the *MAPT* 3′UTR region [22]. In our study, we did not find associations to these *MAPT* variants, but found two other 3′UTR polymorphisms, which were nominally associated with PART. One of these polymorphisms, rs7521, occurs in both haplotypes H1 and H2 [22], which may explain why we did not detect an association with these ancestral haplotypes. In previous analyses on Finns, the *MAPT* variation, including the *H1/H2* haplotype, has been shown to associate only weakly or not at all with different forms of neurodegenerative diseases [58, 59, 60]. The rs7521 polymorphism has been previously associated with Parkinson's disease in a Finnish population, with the *H1/H2* haplotype system not showing any association in the same study [59]. The nominal *MAPT* associations found in the present study were not very strong (lowest *p* value 0.008), and this may have been influenced by the relatively low number of subjects in this study. Furthermore, Bonferroni correction, which was used here, is known to be a very conservative correction method.

The Vantaa 85+ study has many strengths, but also some limitations. Being a prospective population‐based study, the risk of selection bias is minimised. Additionally, the Vantaa 85+ study is well suited to examine PART and other novel neurodegenerative diseases, because this is the age group in which they have been reported to occur at high frequency. On the other hand, the fact that participants have been included according to high age needs to be taken into account when comparing results with those of other studies. The small sample size of the definite PART group (*n* = 16) did not allow us to perform reliable analyses on definite and possible PART separately due to a possible lack of statistical power. Some results of the combined PART group therefore appeared to derive from the possible PART subjects rather than the definite PART subjects, and some of the results of the definite PART group were somewhat divergent from what has usually been reported. Moreover, because especially Braak I–IV/Thal 1–2 subjects (i.e., possible PART) can represent either PART or early/mild AD, this also needs to be taken into account as a possible confounding factor when interpreting the results. Another limitation in our study was that the nuances of cognitive impairment were not studied very precisely in our study, as the categorisation of dementia was only based on the DSM IIIR criteria and only the MMSE test was used to measure the rate of cognitive decline. Lastly, when evaluating Braak stages, it also needs to be pointed out that we did not study the anterior hippocampus, which makes recognition of Braak stage I more difficult as the posterior parts of the hippocampus sampled at the level of the lateral geniculate body only contain remnants of the transentorhinal cortex [32].

In conclusion, our study confirms that many of the previous findings related to PART also apply in a population‐based setting. PART is a common neuropathological finding in the brains of over 85‐year‐olds, and individuals with PART differ from individuals with typical AD‐type neuropathological changes with regard to their pattern of cognitive decline, *APOE* genotype and burden of comorbid pathologies. It is important to separate PART from classical AD and other neurodegenerative disorders in both clinical and neuropathological studies.

## CONFLICT OF INTEREST

Pentti J. Tienari holds a patent on C9orf72 in diagnostics and treatment of ALS/FTD and has made paid consultations to Roche, Biogen, Merck, Teva, Sanofi‐Genzyme and Novartis. Other authors declare no conflicts of interest.

## ETHICS STATEMENT

All participants (or their relatives) have given informed consent for the Vantaa 85+ study. The study was approved by the Ethics Committee of the Health Centre of the City of Vantaa and by the Coordinating Ethics Committee of the Helsinki and Uusimaa Hospital District. The use of the health and social data and death certificates was permitted by the Finnish Health and Social Ministry. The National Authority for Medicolegal Affairs (VALVIRA) approved the collection of tissue samples and their use in research purposes. Next of kin has given consent for each post‐mortem examination.

## AUTHOR CONTRIBUTIONS

Liisa Myllykangas designed and supervised the study. Brain dissection and sampling was mainly performed by Tuomo Polvikoski. Processing and staining of samples were done by Sara Savola, Anna Raunio and Mia Kero. Microscopic studies were carried out by Sara Savola, Liisa Myllykangas and Anders Paetau, and previously also by Tuomo Polvikoski, Mira Mäkelä Anna Raunio and Maarit Tanskanen. Literature search, data analysis and drafting of the manuscript were done by Sara Savola. Genetic analysis was done by Karri Kaivola and Pentti Tienari. Statistical analyses were verified by Jarno Tuimala. Kalle Pärn and Priit Palta performed genotype imputation for the Vantaa 85+ genetic data. All authors participated in the preparation of the manuscript.

### PEER REVIEW

The peer review history for this article is available at https://publons.com/publon/10.1111/nan.12788.

## Supporting information


**Table S1.** Antibodies used in immunohistochemical staining experiments in the present study.
**Table S2.** Results of univariate and multivariate regression analyses to determine predictors of cognitive decline.
**Table S3.** Results of sensitivity analyses done by excluding certain subjects based on their comorbidty pathologies and genetic features. Results are age and sex adjusted.
**Table S4.** Results of logistic regression analysis of the *MAPT 3’*UTR region between the PART and low AD groups using PLINK, adjusted for sex and age.
**Table S5.** Results of logistic regression analysis of the *MAPT 3’*UTR region between the PART and high AD groups using PLINK, adjusted for sex and age.
**Table S6.** Results of Haploview haplotype analysis of the *MAPT 3’*UTR region between the PART and low AD groups.
**Table S7.** Results of Haploview haplotype analysis of the *MAPT 3’*UTR region between the PART and high AD groups.
**Table S8.** Comparison of common *MAPT* haplotypes between the PART and low AD groups.
**Table S9.** Comparison of common *MAPT* haplotypes between the PART and high AD groups.
**Table S10.** Frequency of dementia in the study groups when excluding LATE‐NC with HS, diffuse neocortical and limbic predominant LRP and small cortical infarcts (n = 152 remaining).
**Figure S1.** Line chart showing how MMSE scores changed in follow‐up, using MMSE at baseline in 1991 as the reference. In the background the change in MMSE scores over time are seen for individual study participants, whereas group medians are highlighted.
**Figure S2.** Standardised AD genetic risk scores in the PART, low AD and high AD groups.
**Figure S3.** Standardised AD genetic risk scores without *APOE* in the PART, low AD and high AD groups.

## Data Availability

The data that support the findings of this study are available from the corresponding author upon reasonable request.

## References

[1] Crary JF , Trojanowski JQ , Schneider JA , et al. Primary age‐related tauopathy (PART): a common pathology associated with human aging. Acta Neuropathol. 2014;128(6):755‐766. 25348064 10.1007/s00401-014-1349-0 PMC4257842

[2] Jellinger KA , Attems J . Neurofibrillary tangle‐predominant dementia: comparison with classical Alzheimer disease. Acta Neuropathol. 2007;113(2):107‐117. 17089134 10.1007/s00401-006-0156-7

[3] Noda K , Sasaki K , Fujimi K , et al. Quantitative analysis of neurofibrillary pathology in a general population to reappraise neuropathological criteria for senile dementia of the neurofibrillary tangle type (tangle‐only dementia): the Hisayama study. Neuropathology. 2006;26(6):508‐518. 17203586 10.1111/j.1440-1789.2006.00722.x

[4] Knopman DS , Parisi JE , Salviati A , et al. Neuropathology of cognitively normal elderly. J Neuropathol Exp Neurol. 2003;62(11):1087‐1095. 14656067 10.1093/jnen/62.11.1087

[5] Nelson PT , Abner EL , Schmitt FA , et al. Brains with medial temporal lobe neurofibrillary tangles but no neuritic amyloid plaques are a diagnostic dilemma but may have pathogenetic aspects distinct from Alzheimer disease. J Neuropathol Exp Neurol. 2009;68(7):774‐784. 19535994 10.1097/NEN.0b013e3181aacbe9 PMC2725359

[6] Hyman BT , Phelps CH , Beach TG , et al. National Institute on Aging‐Alzheimer's Association guidelines for the neuropathologic assessment of Alzheimer's disease. Alzheimers Dement. 2012;8(1):1‐13. 22265587 10.1016/j.jalz.2011.10.007 PMC3266529

[7] Montine TJ , Phelps CH , Beach TG , et al. National Institute on Aging‐Alzheimer's Association guidelines for the neuropathologic assessment of Alzheimer's disease: a practical approach. Acta Neuropathol. 2012;123(1):1‐11. 22101365 10.1007/s00401-011-0910-3 PMC3268003

[8] Braak H , Braak E . Neuropathological stageing of Alzheimer‐related changes. Acta Neuropathol. 1991;82(4):239‐259. 1759558 10.1007/BF00308809

[9] Nelson PT , Trojanowski JQ , Abner EL , et al. “New Old Pathologies”: AD, PART, and Cerebral Age‐Related TDP‐43 With Sclerosis (CARTS). J Neuropathol Exp Neurol. 2016;75(6):482‐498. 27209644 10.1093/jnen/nlw033 PMC6366658

[10] Jellinger KA , Alafuzoff I , Attems J , et al. PART, a distinct tauopathy, different from classical sporadic Alzheimer disease. Acta Neuropathol. 2015;129(5):757‐762. 25778618 10.1007/s00401-015-1407-2 PMC4534004

[11] Duyckaerts C , Braak H , Brion JP , et al. PART is part of Alzheimer disease. Acta Neuropathol. 2015;129(5):749‐756. 25628035 10.1007/s00401-015-1390-7 PMC4405349

[12] Braak H , Del Tredici K . Are cases with tau pathology occurring in the absence of Aβ deposits part of the AD‐related pathological process? Acta Neuropathol. 2014;128(6):767‐772. 25359108 10.1007/s00401-014-1356-1

[13] Bell WR , An Y , Kageyama Y , et al. Neuropathologic, genetic, and longitudinal cognitive profiles in primary age‐related tauopathy (PART) and Alzheimer's disease. Alzheimers Dement. 2019;15(1):8‐16. 30465754 10.1016/j.jalz.2018.07.215 PMC6542566

[14] Besser LM , Mock C , Teylan MA , Hassenstab J , Kukull WA , Crary JF . Differences in Cognitive Impairment in Primary Age‐Related Tauopathy Versus Alzheimer Disease. J Neuropathol Exp Neurol. 2019;78(3):219‐228. 30715383 10.1093/jnen/nly132 PMC6380319

[15] Teylan M , Mock C , Gauthreaux K , et al. Cognitive trajectory in mild cognitive impairment due to primary age‐related tauopathy. Brain. 2020;143(2):611‐621. 31942622 10.1093/brain/awz403 PMC7009602

[16] Iida MA , Farrell K , Walker JM , et al. Predictors of cognitive impairment in primary age‐related tauopathy: an autopsy study. Acta Neuropathol Commun. 2021;9(1):134. 34353357 10.1186/s40478-021-01233-3 PMC8340493

[17] Kunkle BW , Grenier‐Boley B , Sims R , et al. Genetic meta‐analysis of diagnosed Alzheimer's disease identifies new risk loci and implicates Aβ, tau, immunity and lipid processing. Nat Genet. 2019;51(3):414‐430. 30820047 10.1038/s41588-019-0358-2 PMC6463297

[18] Wightman DP , Jansen IE , Savage JE , et al. A genome‐wide association study with 1,126,563 individuals identifies new risk loci for Alzheimer's disease. Nat Genet. 2021;53(9):1276‐1282. 34493870 10.1038/s41588-021-00921-z PMC10243600

[19] Robinson AC , Davidson YS , Roncaroli F , et al. Influence of APOE genotype in primary age‐related tauopathy. Acta Neuropathol Commun. 2020;8(1):215. 33287896 10.1186/s40478-020-01095-1 PMC7720601

[20] Corder EH , Saunders AM , Strittmatter WJ , et al. Gene dose of apolipoprotein E type 4 allele and the risk of Alzheimer's disease in late onset families. Science. 1993;261(5123):921‐923. 8346443 10.1126/science.8346443

[21] Corder EH , Saunders AM , Risch NJ , et al. Protective effect of apolipoprotein E type 2 allele for late onset Alzheimer disease. Nat Genet. 1994;7(2):180‐184. 7920638 10.1038/ng0694-180

[22] Santa‐Maria I , Haggiagi A , Liu X , et al. The MAPT H1 haplotype is associated with tangle‐predominant dementia. Acta Neuropathol. 2012;124(5):693‐704. 22802095 10.1007/s00401-012-1017-1 PMC3608475

[23] Walker JM , Richardson TE , Farrell K , et al. Early selective vulnerability of the CA2 hippocampal subfield in primary age‐related tauopathy. J Neuropathol Exp Neurol. 2021;80(2):102‐111. 33367843 10.1093/jnen/nlaa153 PMC8453611

[24] Kovacs GG , Milenkovic I , Wöhrer A , et al. Non‐Alzheimer neurodegenerative pathologies and their combinations are more frequent than commonly believed in the elderly brain: a community‐based autopsy series. Acta Neuropathol. 2013;126(3):365‐384. 23900711 10.1007/s00401-013-1157-y

[25] Robinson JL , Corrada MM , Kovacs GG , et al. Non‐Alzheimer's contributions to dementia and cognitive resilience in the 90+ study. Acta Neuropathol. 2018;136(3):377‐388. 29916037 10.1007/s00401-018-1872-5 PMC6534149

[26] Myllykangas L , Polvikoski T , Sulkava R , et al. Association of lipoprotein lipase Ser447Ter polymorphism with brain infarction: a population‐based neuropathological study. Ann Med. 2001;33(7):486‐492. 11680797 10.3109/07853890109002098

[27] Myllykangas L , Polvikoski T , Sulkava R , et al. Genetic association of alpha2‐macroglobulin with Alzheimer's disease in a Finnish elderly population. Ann Neurol. 1999;46(3):382‐390. 10482269

[28] Barkhof F , Polvikoski TM , van Straaten EC , et al. The significance of medial temporal lobe atrophy: a postmortem MRI study in the very old. Neurology. 2007;69(15):1521‐1527. 17923614 10.1212/01.wnl.0000277459.83543.99

[29] Rahkonen T , Eloniemi‐Sulkava U , Halonen P , et al. Delirium in the non‐demented oldest old in the general population: risk factors and prognosis. Int J Geriatr Psychiatry. 2001;16(4):415‐421. 11333430 10.1002/gps.356

[30] Kero M , Raunio A , Polvikoski T , Tienari PJ , Paetau A , Myllykangas L . Hippocampal sclerosis in the oldest old: a Finnish population‐based study. J Alzheimers Dis. 2018;63(1):263‐272. 29614661 10.3233/JAD-171068 PMC5900558

[31] Braak H , Alafuzoff I , Arzberger T , Kretzschmar H , Del Tredici K . Staging of Alzheimer disease‐associated neurofibrillary pathology using paraffin sections and immunocytochemistry. Acta Neuropathol. 2006;112(4):389‐404. 16906426 10.1007/s00401-006-0127-z PMC3906709

[32] Alafuzoff I , Arzberger T , Al‐Sarraj S , et al. Staging of neurofibrillary pathology in Alzheimer's disease: a study of the BrainNet Europe Consortium. Brain Pathol. 2008;18(4):484‐496. 18371174 10.1111/j.1750-3639.2008.00147.x PMC2659377

[33] Thal DR , Rüb U , Orantes M , Braak H . Phases of A beta‐deposition in the human brain and its relevance for the development of AD. Neurology. 2002;58(12):1791‐1800. 12084879 10.1212/wnl.58.12.1791

[34] Mäkelä M , Paetau A , Polvikoski T , Myllykangas L , Tanskanen M . Capillary amyloid‐β protein deposition in a population‐based study (Vantaa 85+). J Alzheimers Dis. 2016;49(1):149‐157. 26444758 10.3233/JAD-150241

[35] Polvikoski T , Sulkava R , Myllykangas L , et al. Prevalence of Alzheimer's disease in very elderly people: a prospective neuropathological study. Neurology. 2001;56(12):1690‐1696. 11425935 10.1212/wnl.56.12.1690

[36] Tanskanen M , Mäkelä M , Notkola IL , et al. Population‐based analysis of pathological correlates of dementia in the oldest old. Ann Clin Transl Neurol. 2017;4(3):154‐165. 28275649 10.1002/acn3.389 PMC5338150

[37] Raunio A , Kaivola K , Tuimala J , et al. Lewy‐related pathology exhibits two anatomically and genetically distinct progression patterns: a population‐based study of Finns aged 85. Acta Neuropathol. 2019;138(5):771‐782. 31494694 10.1007/s00401-019-02071-3 PMC6800868

[38] Neltner JH , Abner EL , Jicha GA , et al. Brain pathologies in extreme old age. Neurobiol Aging. 2016;37:1‐11. 26597697 10.1016/j.neurobiolaging.2015.10.009 PMC4688098

[39] Braak H , Thal DR , Ghebremedhin E , Del Tredici K . Stages of the pathologic process in Alzheimer disease: age categories from 1 to 100 years. J Neuropathol Exp Neurol. 2011;70(11):960‐969. 22002422 10.1097/NEN.0b013e318232a379

[40] Serrano‐Pozo A , Qian J , Monsell SE , Frosch MP , Betensky RA , Hyman BT . Examination of the clinicopathologic continuum of Alzheimer disease in the autopsy cohort of the National Alzheimer Coordinating Center. J Neuropathol Exp Neurol. 2013;72(12):1182‐1192. 24226270 10.1097/NEN.0000000000000016 PMC3962953

[41] Nelson PT , Alafuzoff I , Bigio EH , et al. Correlation of Alzheimer disease neuropathologic changes with cognitive status: a review of the literature. J Neuropathol Exp Neurol. 2012;71(5):362‐381. 22487856 10.1097/NEN.0b013e31825018f7 PMC3560290

[42] Kawas CH , Kim RC , Sonnen JA , Bullain SS , Trieu T , Corrada MM . Multiple pathologies are common and related to dementia in the oldest‐old: the 90+ study. Neurology. 2015;85(6):535‐542. 26180144 10.1212/WNL.0000000000001831 PMC4540246

[43] Josephs KA , Murray ME , Tosakulwong N , et al. Tau aggregation influences cognition and hippocampal atrophy in the absence of beta‐amyloid: a clinico‐imaging‐pathological study of primary age‐related tauopathy (PART). Acta Neuropathol. 2017;133(5):705‐715. 28160067 10.1007/s00401-017-1681-2 PMC6091858

[44] Rahimi J , Kovacs GG . Prevalence of mixed pathologies in the aging brain. Alzheimers Res Ther. 2014;6(9):82. 25419243 10.1186/s13195-014-0082-1 PMC4239398

[45] Uchikado H , Lin WL , DeLucia MW , Dickson DW . Alzheimer disease with amygdala Lewy bodies: a distinct form of alpha‐synucleinopathy. J Neuropathol Exp Neurol. 2006;65(7):685‐697. 16825955 10.1097/01.jnen.0000225908.90052.07 PMC5706655

[46] Thal DR , Ghebremedhin E , Rüb U , Yamaguchi H , Del Tredici K , Braak H . Two types of sporadic cerebral amyloid angiopathy. J Neuropathol Exp Neurol. 2002;61(3):282‐293. 11895043 10.1093/jnen/61.3.282

[47] Ghebremedhin E , Schultz C , Botez G , et al. Argyrophilic grain disease is associated with apolipoprotein E epsilon 2 allele. Acta Neuropathol. 1998;96(3):222‐224. 9754952 10.1007/s004010050886

[48] Jicha GA , Nelson PT . Hippocampal sclerosis, argyrophilic grain disease, and primary age‐related tauopathy. Continuum (Minneap Minn). 2019;25(1):208‐233. 30707194 10.1212/CON.0000000000000697 PMC6548534

[49] Nelson PT , Dickson DW , Trojanowski JQ , et al. Limbic‐predominant age‐related TDP‐43 encephalopathy (LATE): consensus working group report. Brain. 2019;142(6):1503‐1527. 31039256 10.1093/brain/awz099 PMC6536849

[50] Bancher C , Jellinger KA . Neurofibrillary tangle predominant form of senile dementia of Alzheimer type: a rare subtype in very old subjects. Acta Neuropathol. 1994;88(6):565‐570. 7879604 10.1007/BF00296494

[51] Besser LM , Crary JF , Mock C , Kukull WA . Comparison of symptomatic and asymptomatic persons with primary age‐related tauopathy. Neurology. 2017;89(16):1707‐1715. 28916532 10.1212/WNL.0000000000004521 PMC5644462

[52] Nelson PT , Jicha GA , Schmitt FA , et al. Clinicopathologic correlations in a large Alzheimer disease center autopsy cohort: neuritic plaques and neurofibrillary tangles "do count" when staging disease severity. J Neuropathol Exp Neurol. 2007;66(12):1136‐1146. 18090922 10.1097/nen.0b013e31815c5efb PMC3034246

[53] Arriagada PV , Growdon JH , Hedley‐Whyte ET , Hyman BT . Neurofibrillary tangles but not senile plaques parallel duration and severity of Alzheimer's disease. Neurology. 1992;42(3 Pt 1):631‐639. 1549228 10.1212/wnl.42.3.631

[54] Bancher C , Egensperger R , Kösel S , Jellinger K , Graeber MB . Low prevalence of apolipoprotein E epsilon 4 allele in the neurofibrillary tangle predominant form of senile dementia. Acta Neuropathol. 1997;94(5):403‐409. 9386771 10.1007/s004010050726

[55] Ikeda K , Akiyama H , Arai T , et al. A subset of senile dementia with high incidence of the apolipoprotein E epsilon2 allele. Ann Neurol. 1997;41(5):693‐695. 9153535 10.1002/ana.410410522

[56] Teylan M , Besser LM , Crary JF , et al. Clinical diagnoses among individuals with primary age‐related tauopathy versus Alzheimer's neuropathology. Lab Invest. 2019;99(7):1049‐1055. 30710118 10.1038/s41374-019-0186-0 PMC6609478

[57] Polvikoski T , Sulkava R , Haltia M , et al. Apolipoprotein E, dementia, and cortical deposition of beta‐amyloid protein. N Engl J Med. 1995;333(19):1242‐1247. 7566000 10.1056/NEJM199511093331902

[58] Kaivorinne AL , Krüger J , Kuivaniemi K , et al. Role of MAPT mutations and haplotype in frontotemporal lobar degeneration in Northern Finland. BMC Neurol. 2008;8(1):48. 19091059 10.1186/1471-2377-8-48 PMC2625345

[59] Fung HC , Xiromerisiou G , Gibbs JR , et al. Association of tau haplotype‐tagging polymorphisms with Parkinson's disease in diverse ethnic Parkinson's disease cohorts. Neurodegener Dis. 2006;3(6):327‐333. 17192721 10.1159/000097301

[60] Baker M , Graff‐Radford D , Wavrant DeVrièze F , et al. No association between TAU haplotype and Alzheimer's disease in population or clinic based series or in familial disease. Neurosci Lett. 2000;285(2):147‐149. 10793248 10.1016/s0304-3940(00)01057-0

