# Supplementary material for: Primary age‐related tauopathy in a Finnish population‐based study of the oldest old (Vantaa 85+)
Source: Neuropathol Appl Neurobiol. 2022 Jan 23;48(3):e12788. doi: 10.1111/nan.12788 (PMC9305229; doi:10.1111/nan.12788)
Supplement: Supplementary file 1 — Table S1. Antibodies used in immunohistochemical staining experiments in the present study. Table S2. Results of univariate and multivariate regression analyses to determine predictors of cognitive decline. Table S3. Results of sensitivity analyses done by excluding certain subjects based on their comorbidty pathologies and genetic features. Results are age and sex adjusted. Table S4. Results of logistic regression analysis of the MAPT 3’UTR region between the PART and low AD groups using PLINK, adjusted for sex and age. Table S5. Results of logistic regression analysis of the MAPT 3’UTR region between the PART and high AD groups using PLINK, adjusted for sex and age. Table S6. Results of Haploview haplotype analysis of the MAPT 3’UTR region between the PART and low AD groups. Table S7. Results of Haploview haplotype analysis of the MAPT 3’UTR region between the PART and high AD groups. Table S8. Comparison of common MAPT haplotypes between the PART and low AD groups. Table S9. Comparison of common MAPT haplotypes between the PART and high AD groups. Table S10. Frequency of dementia in the study groups when excluding LATE‐NC with HS, diffuse neocortical and limbic predominant LRP and small cortical infarcts (n = 152 remaining). Figure S1. Line chart showing how MMSE scores changed in follow‐up, using MMSE at baseline in 1991 as the reference. In the background the change in MMSE scores over time are seen for individual study participants, whereas group medians are highlighted. Figure S2. Standardised AD genetic risk scores in the PART, low AD and high AD groups. Figure S3. Standardised AD genetic risk scores without APOE in the PART, low AD and high AD groups. [file NAN-48-0-s001.docx]

**Supplementary material**

Article type: Original Article

**Title**

Primary Age-Related Tauopathy (PART) in a Finnish Population-Based Study of the Oldest Old (Vantaa 85+)

**Authors**

Sara Savola^ab^, Karri Kaivola^cd^, Anna Raunio^ab^, Mia Kero^ab^, Mira Mäkelä^ab^, Kalle Pärn^e^, Priit Palta^e^, Maarit Tanskanen^ab^, Jarno Tuimala^a^, Tuomo Polvikoski^f^, Pentti J. Tienari^cd^, Anders Paetau^ab^, and Liisa Myllykangas^ab^

**Affiliations**

^a^Department of Pathology, University of Helsinki, Helsinki, Finland

^b^Department of Pathology, HUS Diagnostic Center, Helsinki University Hospital, Helsinki, Finland

^c^Translational Immunology, Research Programs Unit, University of Helsinki, Helsinki, Finland

^d^Department of Neurology, University of Helsinki and Helsinki University Hospital, Helsinki, Finland

^e^Institute for Molecular Medicine Finland (FIMM), HiLIFE, University of Helsinki, Helsinki, Finland

^f^Translational and Clinical Research Institute, Newcastle University, Newcastle upon Tyne, United Kingdom

**Corresponding author**

Liisa Myllykangas, MD, PhD

Department of Pathology

PO Box 21 (Haartmaninkatu 3)

00014 University of Helsinki

Email: [liisa.myllykangas@helsinki.fi](mailto:liisa.myllykangas@helsinki.fi)

10 supplementary tables, 3 supplementary figures

Supplementary Methods

Genetic analysis of the *MAPT* 3′UTR region

We performed genome-wide genotyping for 512 participants with the HumanCNV370 array (Illumina, CA, USA) according to the manufacturer’s recommendations (1). BeadStudio v. 3.2 (Illumina) was used for reclustering with a no-call threshold of 0.15. Samples then underwent standard pre-imputation quality control and were imputed with the Finnish population-specific SISu v3 imputation reference panel by using Beagle 4.1. The imputation protocol is described in more detail at: dx.doi.org/10.17504/protocols.io.nmndc5e.  Post-imputation quality control consisted of discarding variants with an imputation INFO score < 0.3 and a minor allele frequency threshold of 0.01. We retrieved *MAPT*3'UTR genomic coordinates from UCSC table browser (chr17:46024171:46028334, hg38).

There remained 29 variants in 58 individuals with PART, 111 individuals with low AD-type neuropathological changes and 95 individuals with high AD-type neuropathological changes for association analyses. We used PLINK 1.9 to perform logistic regression analysis of the *MAPT* *3'*UTR region with age and sex as covariates ​to compare 1) individuals with PART and individuals with low AD-type neuropathological changes and 2) individuals with PART and individuals with high AD-type neuropathological changes. We also performed a haplotype analysis between PART and AD-type neuropathological changes groups. Haplotypes were constructed and analysed using Haploview's default settings from 15 SNPs in the *MAPT 3'*UTR region. We also tested the association of the previously reported (2) *MAPT* haplotypes with the same phenotypes.

Calculation of AD genetic risk scores

We calculated Alzheimer’s disease (AD) genetic risk scores (GRS) for 505 samples with imputed genotype data in the Vantaa85+ cohort. The GRS calculations were based on 21 genome-wide significant and replicable variants from a large genome-wide association meta-analysis on AD (3). The raw AD GRS scores were calculated by summing the effect alleles weighted by their beta value (natural logarithm of odds ratio) as implemented in PLINK v.1.9. Of the 21 variants, only the *TREM2*variant rs75932628 was not present in the data due to its rarity. The samples were then divided into three neuropathological groups: PART, low AD-type neuropathological changes, and high AD-type neuropathological changes. Other samples were used as control samples and were used to transform raw GRS scores into standard scores (z-scores). Since *APOE* ε4 is known as the strongest genetic risk factor for AD (4), we also calculated GRS without *APOE*.

We identified four individuals as outliers (>1.5 times the interquartile range above the third quartile or below the first quartile) in the PART group: three with high AD GRS and one individual with low AD GRS. For the sensitivity analysis these four individuals were excluded.

Scoring of argyrophilic grains

Argyrophilic grains were detected by using 80 μm thick sections cut from polyethylene glycol-embedded samples containing the (1) entorhinal and transentorhinal cortex at coronal level of the mammillary bodies and (2) hippocampus with neighboring basal temporal cortex at coronal level of the lateral geniculate body. The free-floating sections were stained with Gallyas silver method. Grains were identified as short comma- and/or rod-like silver-positive particles within the neuropil. When present, the grains were seen widely in the upper layers of the cortex. Scoring was based on semi-quantitative assessment of frequency of the grains and extent of the most severe grain pathology: 0 = no grains detected, 1 = uncertain grain-like structures in one sample, 2 = uncertain grain-like structures in two samples, 3 = grains of moderate frequency in one sample, 4 = grains of moderate frequency in two samples, 5 = high frequency of grains in one sample and 6 = high frequency of grains in two samples. For simplification purposes, we combined these stages into two groups as follows: stages 0-2 were coded as negative, and stages 3-6 as positive.

Supplementary Results

Results of the *MAPT 3’*UTR region analysis

In the analyses of the *MAPT 3’*UTR region, there were no statistically significant differences between the groups after Bonferroni correction (Suppl. Tables 4-7). Nominally significant associations (0.01 < *p* < 0.05) were detected with two *MAPT* 3*′*UTR polymorphisms in comparison between PART versus the low AD group (Suppl. Table 4), but they did not survive Bonferroni correction. The haplotype analysis identified two nominally significant haplotypes when comparing PART and low AD, and one nominally significant haplotype when comparing PART and high AD. However, these results did not survive Bonferroni correction (Suppl. Table 6-7). No associations were seen in analysis of previously reported common *MAPT* haplotypes (Suppl. Tables 8-9) (2). However, it needs to be noted that the statistical power of our sample size might not have been enough to show associations.

Results of AD GRS analysis

Standardised AD GRS of our participants are visualised groupwise in Supplementary figures 2 and 3. The burden of AD genetic risk variants was highest in the high AD group and lowest in the PART group. The high AD group had a median z-score of 0.81, the low AD group had a median z-score of -0.27 and the PART group had a median z-score of -0.50 (Suppl. figure 2). When excluding *APOE*, the differences in AD GRS decreased but the GRS remained highest in the high AD group and lowest in the PART group. The high AD group had a median z-score of 0.12, the low AD group had a median z-score of -0.020 and PART group had a median z-score of -0.18 (Suppl. figure 3).

Supplementary Table 1. Antibodies used in immunohistochemical staining experiments in the present study.

| Antibody | Clone | Host species, antibody type | Catalog number, manufacturer | Concentration |
| --- | --- | --- | --- | --- |
| Phospho-Tau (Ser202, Thr205) | AT8 | Mouse, monoclonal | MN-1020, Thermofisher/Invitrogen, USA | 200 ng/ml |
| Anti-β-amyloid, residues 17-24 | 4G8 | Mouse, monoclonal | 800701, BioLegend, San Diego, CA, USA | 70 ng/ml |

^1^References to the previously published data are found in the Material and Methods.

Supplementary Table 2. Results of univariate and multivariate regression analyses to determine predictors of cognitive decline.

OR, odds ratio. CI, confidence interval. B, unstandardized coefficient B. ***p- values  < 0.001, **p-values < 0.01, *p-values < 0.05, ^(^*^)^p-values < 0.1.

^†^We performed both univariate (age and sex adjusted) and multivariate binary logistic regression analyses with dementia (yes/no) as the dependent variable and the variables seen in the leftmost column as independent variables.

^††^ We performed both univariate (age and sex adjusted) and multivariate linear regression analyses with MMSE as the dependent variable and the variables seen in the leftmost column as independent variables.

| Independent variable | ^†^Dementia  OR (95% CI) *p*-value | ^††^MMSE -91  B (95% CI) *p*-value | ^††^Change in MMSE -91 vs. -94  B (95% CI) *p*-value | ^††^Change in MMSE -91 vs. -96  B (95% CI) *p*-value |
| --- | --- | --- | --- | --- |
| Univariate analysis  age and sex adjusted only |  |  |  |  |
| PART vs. low AD | 1.36 (0.74 to 2.51) 0.326 | -0.34 (-2.98 to 2.30) 0.802 | -2.69 (-4.76 to -0.61) **0.012*** | -4.00 (-7.52 to -0.48) **0.026*** |
| PART vs. high AD | 8.04 (3.70 to 17.48) **<0.001***** | -6.22 (-8.99 to -3.45) **<0.001***** | -3.59 (-5.83 to -1.36) **0.002**** | -5.33 (-9.10 to -1.55) **0.006**** |
| Age in -91 | 1.05 (0.97 to 1.14) 0.252 | -0.66 (-0.99 to -0.34) **<0.001***** | -0.20 (-0.49 to 0.08) 0.163 | -0.12 (-0.60 to 0.37) 0.630 |
| sex | 1.53 (0.78 to 2.99) 0.219 | -4.63 (-7.38 to -1.88) **0.001**** | -0.08 (-3.00 to 0.99) 0.322 | -2.09 (-5.47 to 1.28) 0.221 |
| Multivariate analysis |  |  |  |  |
| PART vs. low AD | 1.24 (0.62 to 2.51) 0.547 | 0.28 (-2.51 to 3.06) 0.846 | -2.99 (-5.45 to -0.53) **0.018*** | -3.30 (-7.34 to 0.74) 0.107 |
| PART vs. high AD | 6.99 (2.93 to 16.71) **<0.001***** | -4.66 (-7.65 to -1.67) **0.002**** | -3.96 (-6.66 to -1.26) **0.004**** | -4.23 (-8.87 to 0.41) **0.073^(^*^)^** |
| Age in -91 | 1.02 (0.92 to 1.12) 0.760 | -0.52 (-0.89 to -0.16) **0.005**** | -0.19 (-0.54 to 0.16) 0.280 | -0.07 (-0.64 to 0.50) 0.811 |
| sex | 1.27 (0.59 to 2.77) 0.544 | -4.60 (-7.48 to -1.73) **0.002**** | -0.75 (-3.13 to 1.64) 0.536 | -2.50 (-6.90 to 1.90) 0.260 |
| LATE-NC with HS | 11.19 (2.51 to 49.94) **0.002**** | -4.68 (-7.65 to -1.71) **0.002**** | -0.70 (-3.50 to 2.10) 0.622 | -1.33 (-5.38 to 2.73) 0.516 |
| AGD | 1.20 (0.62 to 2.32) 0.593 | 0.05 (-2.36 to 2.46) 0.967 | 0.73 (-1.46 to 2.92) 0.508 | -0.30 (-3.76 to 3.15) 0.861 |
| DLB: diffuse neo-cortical and limbic predominant vs. no LRP | 2.58 (1.30 to 5.13) **0.007**** | -2.63 (-4.98 to -0.28) **0.029*** | -1.12 (-3.40 to 1.17) 0.334 | -2.78 (-6.41 to 0.86) 0.132 |
| Small cortical infarct  (2-15 mm) | 1.72 (0.99 to 3.01) **0.055^(^*^)^** | -1.79 (-3.24 to -0.35) **0.015*** | 0.36 (-1.06 to 1.78) 0.618 | 0.02 (-2.40 to 2.44) 0.987 |

Supplementary Table 3. Results of sensitivity analyses done by excluding certain subjects based on their comorbidty pathologies and genetic features. Results are age and sex adjusted.

|  | ^†^Dementia  OR (95% CI) *p*-value | ^††^MMSE -91  B (95% CI) *p*-value | ^††^Change in MMSE -91 vs. -94  B (95% CI) *p*-value | ^††^Change in MMSE -91 vs. -96  B (95% CI) *p*-value |
| --- | --- | --- | --- | --- |
| Excluding diffuse neocortical and limbic predominant LRP |  |  |  |  |
| PART vs. low AD | 1.25 (0.63 to 2.51) 0.524 | -0.26 (-3.17 to 2.66) 0.862 | -2.89 (-5.35 to -0.42) **0.022*** | -2.84 (-6.30 to 0.63) 0.107 |
| PART vs. high AD | 7.24 (2.97 to 17.64) **<0.001***** | -5.30 (-8.48 to -2.12) **0.001**** | -4.22 (-6.89 to -1.54) **0.002**** | -6.37 (-10.21 to -2.52) **0.002**** |
| age in -91 | 1.05 (0.95 to 1.16) 0.344 | -0.67 (-1.06 to -0.28) **0.001**** | -0.27 (-0.62 to 0.07) 0.118 | 0.18 (-0.34 to .69) 0.488 |
| Sex | 1.40 (0.61 to 3.18) 0.425 | -3.14 (-6.41 to 0.13) 0.060 | -0.80 (-3.33 to 1.73) 0.530 | 0.35 (-3.52 to 4.23) 0.856 |
| Excluding LATE-NC with HS |  |  |  |  |
| PART vs. low AD | 1.27 (0.66 to 2.42) 0.474 | 0.13 (-2.64 to 2.90) 0.928 | -2.41 (-4.64 to -0.19) **0.033*** | -3.56 (-7.27 to 0.16) 0.061 |
| PART vs. high AD | 7.08 (3.19 to 15.72) **<0.001***** | -5.35 (-8.31 to -2.40) **<0.001***** | -3.77 (-6.19 to -1.35) **0.003**** | -4.36 (-8.44 to -0.28) **0.037*** |
| age in -91 | 1.03 (0.94 to 1.13) 0.510 | -0.60 (-0.96 to -0.24) **0.001**** | -0.19 (-0.51 to 0.12) 0.225 | -0.08 (-0.59 to 0.43) 0.748 |
| sex | 1.26 (0.63 to 2.54) 0.517 | -4.15 (-7.05 to -1.24) **0.005**** | -1.28 (-3.42 to 0.86) 0.238 | -2.78 (-6.42 to 0.86) 0.132 |
| Excluding AGD |  |  |  |  |
| PART vs. low AD | 1.67 (0.78 to 3.56) 0.189 | 0.12 (-3.04 to 3.28) 0.939 | -3.20 (-6.07 to -0.32) **0.030*** | -5.83 (-10.12 to -1.54) **0.009**** |
| PART vs. high AD | 10.79 (4.29 to 27.14) **<0.001***** | -7.12 (-10.30 to -3.93) **<0.001***** | -4.08 (-7.09 to -1.07) **0.009**** | -6.87 (-11.27 to -2.46) **0.003**** |
| age in -91 | 1.04 (0.95 to 1.15) 0.379 | -0.66 (-1.02 to -0.29) **0.001**** | -0.17 (-0.56 to 0.21) 0.372 | -0.10 (-0.66 to 0.47) 0.734 |
| sex | 1.89 (0.86 to 4.18) 0.114 | -5.38 (-8.50 to -2.27) **0.001**** | -1.10 (-3.68 to 1.47) 0.397 | -2.51 (-6.16 to 1.13) 0.173 |
| Excluding small cortical infarcts  (2-15 mm) |  |  |  |  |
| PART vs. low AD | 1.33 (0.68 to 2.59) 0.399 | -0.25 (-3.12 to 2.62) 0.863 | -3.03 (-5.40 to -0.66) **0.013*** | -3.22 (-6.79 to 0.35) 0.076 |
| PART vs. high AD | 8.71 (3.76 to 20.16) **<0.001***** | -6.10 (-9.09 to -3.11) **<0.001***** | -4.25 (-6.84 to -1.66) **0.002**** | -5.51 (-9.46 to -1.56) **0.007**** |
| Age in -91 | 1.03 (.94 to 1.12) 0.515 | -0.65 (-1.01 to -0.29) **<0.001***** | -0.26 (-0.59 to 0.08) 0.130 | -0.04 (-0.56 to 0.48) 0.870 |
| sex | 1.31 (0.62 to 2.78) 0.478 | -4.38 (-7.44 to -1.32) **0.005**** | -1.30 (-3.65 to 1.05) 0.276 | -1.66 (-5.31 to 2.00) 0.369 |
| Excluding severe CAA^a^ |  |  |  |  |
| PART vs. low AD | 1.32 (0.70 to 2.50) 0.397 | -0.29 (-3.05 to 2.47) 0.836 | -2.75 (-5.03 to -0.46) **0.019*** | -3.24 (-6.68 to 0.19) 0.064 |
| PART vs. high AD | 5.96 (2.47 to 14.42) **<0.001***** | -5.05 (-8.23 to -1.86) **0.002**** | -4.78 (-7.66 to -1.91) **0.001**** | -5.52 (-10.03 to -1.00) **0.018*** |
| Age in -91 | 1.08 (0.99 to 1.18) 0.097 | -0.64 (-1.00 to -0.28) **<0.001***** | -0.22 (-0.56 to 0.12) 0.210 | 0.05 (-0.49 to 0.58) 0.864 |
| sex | 1.15 (0.51 to 2.58) 0.734 | -4.49 (-7.90 to -1.08) **0.010*** | -1.13 (-3.67 to 1.41) 0.380 | -3.63 (-7.77 to 0.51) 0.085 |
| Excluding PART group AD GRS outliers |  |  |  |  |
| PART vs. low AD | 1.36 (.73 to 2.55) 0.333 | -0.40 (-3.09 to 2.29) 0.768 | -2.52 (-4.61 to -0.42) **0.019*** | -3.91 (-7.53 to -0.30) **0.034*** |
| PART vs. high AD | 8.06 (3.67 to 17.71) **<0.001***** | -6.29 (-9.11 to -3.47) **<0.001***** | -3.41 (-5.67 to -1.16) **0.003**** | -5.23 (-9.10 to -1.36) **0.009**** |
| Age in -91 | 1.05 (0.97 to 1.14) 0.226 | -0.68 (-1.00 to -0.35) **<0.001***** | -0.20 (-0.49 to 0.09) 0.165 | -0.12 (-0.61 to 0.37) 0.631 |
| sex | 1.44 (0.73 to 2.85) 0.296 | -4.39 (-7.17 to -1.61) **0.002**** | -1.07 (-3.06 to 0.93) 0.293 | -2.11 (-5.52 to 1.29) 0.220 |
| Excluding *APOE* ε2 and ε4 (i.e., *APOE* genotype ε3ε3 remaining) |  |  |  |  |
| PART vs. low AD | 1.11 (0.52 to 2.35) 0.786 | 0.47 (-2.72 to 3.65) 0.773 | -2.43 (-4.71 to -0.14) **0.038*** | -2.68 (-6.65 to 1.29) 0.181 |
| PART vs. high AD | 4.15 (1.56 to 11.08) **0.004**** | -3.39 (-7.09 to 0.31) **0.072** | -2.85 (-5.52 to -0.18) **0.036*** | -3.47 (-8.35 to 1.42) 0.160 |
| Age in -91 | 1.02 (0.92 to 1.14) 0.683 | -0.55 (-0.97 to -0.12) **0.013*** | -0.24 (-0.58 to 0.10) 0.165 | -1.96 (-7.05 to 3.14) 0.443 |
| sex | 1.44 (0.50 to 4.18) 0.499 | -3.79 (-8.22 to 0.64) 0.093 | -1.44 (-4.25 to 1.37) 0.310 | 0.31 (-0.31 to 0.93) 0.313 |

OR, odds ratio. CI, confidence interval. B, unstandardised coefficient B. ***p- values  < 0.001, **p-values < 0.01, *p-values < 0.05.

^†^We excluded certain subjects based on their comorbidity pathologies and genetic features, and then performed binary logistic regression analysis (age and sex adjusted) with dementia (yes/no) as the dependent variable and PART vs. low/high AD as the independent variable.

^††^We excluded certain subjects based on their comorbidity pathologies and genetic features, and then performed linear regression analysis (age and sex adjusted) with MMSE as the dependent variable and PART vs. low/high AD as the independent variable.

^a^Severity of CAA has been assessed previously (5) by counting the percentage of Aβ stained blood vessels in six brain regions (frontal, parietal, temporal and occipital lobes, hippocampus and cerebellum).Values higher than the upper quartile (5.3 %) were defined as severe CAA.

Supplementary Table 4. Results of logistic regression analysis of the *MAPT* *3'*UTR region between the PART and low AD groups using PLINK, adjusted for sex and age.

| CHR | variant | BP (hg38) | A1 | TEST | n | OR | STAT | *p* |
| --- | --- | --- | --- | --- | --- | --- | --- | --- |
| 17 | rs7521 | 46028029 | G | ADD | 169 | 0.60 | -2.391 | **0.017** |
| 17 | rs564954259 | 46024235 | G | ADD | 169 | 2.94 | 2.18 | **0.029** |
| 17 | rs5820605 | 46025316 | CT | ADD | 169 | 0.70 | -1.539 | 0.124 |
| 17 | rs11331969 | 46027246 | TG | ADD | 169 | 0.69 | -1.441 | 0.150 |
| 17 | rs17574005 | 46024635 | A | ADD | 169 | 1.46 | 0.8266 | 0.409 |
| 17 | rs181844055 | 46027127 | C | ADD | 169 | 0.45 | -0.6951 | 0.487 |
| 17 | rs2158256 | 46026981 | A | ADD | 169 | 0.63 | -0.548 | 0.584 |
| 17 | rs16940802 | 46026338 | A | ADD | 169 | 0.63 | -0.3939 | 0.694 |
| 17 | chr17:46027210 | 46027210 | C | ADD | 169 | 0.88 | -0.3298 | 0.742 |
| 17 | rs9468 | 46024197 | C | ADD | 169 | 0.92 | -0.2149 | 0.830 |
| 17 | chr17:46024409 | 46024409 | AT | ADD | 169 | 0.92 | -0.2149 | 0.830 |
| 17 | chr17:46024483 | 46024483 | TC | ADD | 169 | 0.92 | -0.2149 | 0.830 |
| 17 | rs8712 | 46024505 | G | ADD | 169 | 0.92 | -0.2149 | 0.830 |
| 17 | rs141412361 | 46025077 | CCT | ADD | 169 | 0.92 | -0.2028 | 0.839 |
| 17 | rs1052587 | 46025238 | C | ADD | 169 | 0.92 | -0.2028 | 0.839 |
| 17 | rs1052590 | 46025272 | G | ADD | 169 | 0.92 | -0.2028 | 0.839 |
| 17 | rs1052594 | 46025323 | C | ADD | 169 | 0.92 | -0.2028 | 0.839 |
| 17 | chr17:46025375 | 46025375 | T | ADD | 169 | 0.92 | -0.2028 | 0.839 |
| 17 | rs17574040 | 46025499 | C | ADD | 169 | 0.92 | -0.2028 | 0.839 |
| 17 | rs16940799 | 46025567 | C | ADD | 169 | 0.92 | -0.2028 | 0.839 |
| 17 | rs7687 | 46025930 | C | ADD | 169 | 0.92 | -0.2028 | 0.839 |
| 17 | rs17652748 | 46026250 | T | ADD | 169 | 0.92 | -0.2028 | 0.839 |
| 17 | rs75010486 | 46026459 | C | ADD | 169 | 0.92 | -0.2028 | 0.839 |
| 17 | rs16940806 | 46026460 | A | ADD | 169 | 0.92 | -0.2028 | 0.839 |
| 17 | chr17:46026561 | 46026561 | CT | ADD | 169 | 0.92 | -0.2028 | 0.839 |
| 17 | chr17:46026912 | 46026912 | T | ADD | 169 | 0.92 | -0.2028 | 0.839 |
| 17 | rs2158257 | 46026977 | C | ADD | 169 | 0.92 | -0.2028 | 0.839 |
| 17 | chr17:46027044 | 46027044 | T | ADD | 169 | 0.92 | -0.2028 | 0.839 |
| 17 | rs17574228 | 46027143 | C | ADD | 169 | 0.92 | -0.2028 | 0.839 |

CHR, Chromosome. BP, base pair. A1, effect allele (minor allele). TEST, statistical model used (additive model). STAT, t distribution.

Supplementary Table 5. Results of logistic regression analysis of the *MAPT* *3'*UTR region between the PART and high AD groups using PLINK, adjusted for sex and age.

| CHR | variant | BP (hg38) | A1 | TEST | n | OR | STAT | *p* |
| --- | --- | --- | --- | --- | --- | --- | --- | --- |
| 17 | rs7521 | 46028029 | G | ADD | 153 | 0.65 | -1.768 | 0.077 |
| 17 | rs564954259 | 46024235 | G | ADD | 153 | 2.35 | 1.743 | 0.081 |
| 17 | rs11331969 | 46027246 | TG | ADD | 153 | 0.67 | -1.532 | 0.126 |
| 17 | rs5820605 | 46025316 | CT | ADD | 153 | 0.74 | -1.183 | 0.237 |
| 17 | rs181844055 | 46027127 | C | ADD | 153 | 0.30 | -1.072 | 0.284 |
| 17 | rs16940802 | 46026338 | A | ADD | 153 | 0.35 | -1.032 | 0.302 |
| 17 | rs141412361 | 46025077 | CCT | ADD | 153 | 1.28 | 0.586 | 0.558 |
| 17 | rs1052587 | 46025238 | C | ADD | 153 | 1.28 | 0.586 | 0.558 |
| 17 | rs1052590 | 46025272 | G | ADD | 153 | 1.28 | 0.586 | 0.558 |
| 17 | rs1052594 | 46025323 | C | ADD | 153 | 1.28 | 0.586 | 0.558 |
| 17 | chr17:46025375 | 46025375 | T | ADD | 153 | 1.28 | 0.586 | 0.558 |
| 17 | rs17574040 | 46025499 | C | ADD | 153 | 1.28 | 0.586 | 0.558 |
| 17 | rs16940799 | 46025567 | C | ADD | 153 | 1.28 | 0.586 | 0.558 |
| 17 | rs7687 | 46025930 | C | ADD | 153 | 1.28 | 0.586 | 0.558 |
| 17 | rs17652748 | 46026250 | T | ADD | 153 | 1.28 | 0.586 | 0.558 |
| 17 | rs75010486 | 46026459 | C | ADD | 153 | 1.28 | 0.586 | 0.558 |
| 17 | rs16940806 | 46026460 | A | ADD | 153 | 1.28 | 0.586 | 0.558 |
| 17 | chr17:46026561 | 46026561 | CT | ADD | 153 | 1.28 | 0.586 | 0.558 |
| 17 | chr17:46026912 | 46026912 | T | ADD | 153 | 1.28 | 0.586 | 0.558 |
| 17 | rs2158257 | 46026977 | C | ADD | 153 | 1.28 | 0.586 | 0.558 |
| 17 | chr17:46027044 | 46027044 | T | ADD | 153 | 1.28 | 0.586 | 0.558 |
| 17 | rs17574228 | 46027143 | C | ADD | 153 | 1.28 | 0.586 | 0.558 |
| 17 | chr17:46027210 | 46027210 | C | ADD | 153 | 1.28 | 0.586 | 0.558 |
| 17 | rs9468 | 46024197 | C | ADD | 153 | 1.23 | 0.5129 | 0.608 |
| 17 | chr17:46024409 | 46024409 | AT | ADD | 153 | 1.23 | 0.5129 | 0.608 |
| 17 | chr17:46024483 | 46024483 | TC | ADD | 153 | 1.23 | 0.5129 | 0.608 |
| 17 | rs8712 | 46024505 | G | ADD | 153 | 1.23 | 0.5129 | 0.608 |
| 17 | rs17574005 | 46024635 | A | ADD | 153 | 1.16 | 0.3276 | 0.743 |
| 17 | rs2158256 | 46026981 | A | ADD | 153 | 0.86 | -0.1618 | 0.872 |

CHR, Chromosome. BP, base pair. A1, effect allele (minor allele). TEST, statistical model used (additive model). STAT, t distribution.

Supplementary Table 6. Results of Haploview haplotype analysis of the *MAPT* *3'*UTR region between the PART and low AD groups.

| Haplotype^a^ | Total freq. | Case freq. | Control freq. | χ2 | *p* |
| --- | --- | --- | --- | --- | --- |
| TAGTAGATTCTGATA | 0.445 | 0.534 | 0.405 | 5.13 | **0.024** |
| TAGTAGATTCTGATG | 0.406 | 0.284 | 0.432 | 7.07 | **0.008** |
| CGGCGCCCCTCACCG | 0.08 | 0.095 | 0.099 | 0.02 | 0.9 |
| TAATAGATTCTGATA | 0.058 | 0.078 | 0.054 | 0.72 | 0.395 |
| CGGTAGATTCTGATG | 0.011 | 0.009 | 0.009 | 0.001 | 0.971 |

^a^Haplotypes defined by the following variants: rs9468, rs8712, rs17574005, rs1052587, rs1052590, rs1052594, rs17574040, rs16940799, rs7687, rs17652748, rs75010486, rs16940806, rs2158257, rs17574228, rs7521.

Supplementary Table 7. Results of Haploview haplotype analysis of the *MAPT* *3'*UTR region between the PART and high AD groups.

| Haplotype^a^ | Total freq. | Case freq. | Control freq. | χ2 | *p* |
| --- | --- | --- | --- | --- | --- |
| TAGTAGATTCTGATA | 0.445 | 0.534 | 0.447 | 2.19 | 0.139 |
| TAGTAGATTCTGATG | 0.406 | 0.284 | 0.4 | 4.19 | **0.041** |
| CGGCGCCCCTCACCG | 0.08 | 0.095 | 0.074 | 0.43 | 0.512 |
| TAATAGATTCTGATA | 0.058 | 0.078 | 0.068 | 0.09 | 0.763 |
| CGGTAGATTCTGATG | 0.011 | 0.009 | 0.011 | 0.03 | 0.870 |

^a^Haplotypes defined by the following variants: rs9468, rs8712, rs17574005, rs1052587, rs1052590, rs1052594, rs17574040, rs16940799, rs7687, rs17652748, rs75010486, rs16940806, rs2158257, rs17574228, rs7521.

Supplementary Table 8. Comparison of common *MAPT* haplotypes between the PART and low AD groups.

| Name | Haplotype^a^ | Freq. | Case-Control Freq. | χ2 | *p* |
| --- | --- | --- | --- | --- | --- |
| H1b | GGGCTA | 0.141 | 0.177, 0.105 | 3.448 | 0.0633 |
| H1c | AAGTTG | 0.137 | 0.115, 0.120 | 0.018 | 0.8946 |
| H1d | AAGCTA | 0.132 | 0.177, 0.120 | 2.038 | 0.1534 |
| H2a | AGGCCG | 0.08 | 0.102, 0.095 | 0.035 | 0.8507 |
| H1e | AGGCTA | 0.054 | 0.051, 0.063 | 0.211 | 0.646 |

^a^Haplotypes defined by the following variants as previously reported (2): rs1467967, rs242557, rs3785883, rs2471738, rs9468, rs7521.

Supplementary Table 9. Comparison of common *MAPT* haplotypes between the PART and high AD groups.

| Name | Haplotype^a^ | Freq. | Case-Control Freq. | χ2 | *p* |
| --- | --- | --- | --- | --- | --- |
| H1b | GGGCTA | 0.141 | 0.177, 0.182 | 0.015 | 0.9025 |
| H1c | AAGTTG | 0.137 | 0.115, 0.126 | 0.074 | 0.7861 |
| H1d | AAGCTA | 0.132 | 0.177, 0.125 | 1.569 | 0.2103 |
| H2a | AGGCCG | 0.080 | 0.102, 0.073 | 0.797 | 0.3719 |
| H1e | AGGCTA | 0.054 | 0.051, 0.054 | 0.011 | 0.9181 |

^a^Haplotypes defined by the following variants as previously reported (2): rs1467967, rs242557, rs3785883, rs2471738, rs9468, rs7521.

Supplementary Table 10. Frequency of dementia in the study groups when excluding LATE-NC with HS, diffuse neocortical and limbic predominant LRP and small cortical infarcts (n=152 remaining).

|  | All PART  N=39 | Definite  PART  N= 12 | Possible  PART  N=26 | Low AD  N=70 | High AD  N=43 | *p*  all PART vs low AD | *p*  all PART vs high AD |
| --- | --- | --- | --- | --- | --- | --- | --- |
| Dementia^a^  n(%) | 14 (35.9) | 6 (46.2) | 8 (30.8) | 26 (37.1) | 34 (79.1) | NS | <0.001 |

^a^Results are age and sex adjusted.

Supplementary Figure 1. Line chart showing how MMSE scores changed in follow-up, using MMSE at baseline in 1991 as the reference. In the background the change in MMSE scores over time are seen for individual study participants, whereas group medians are highlighted.


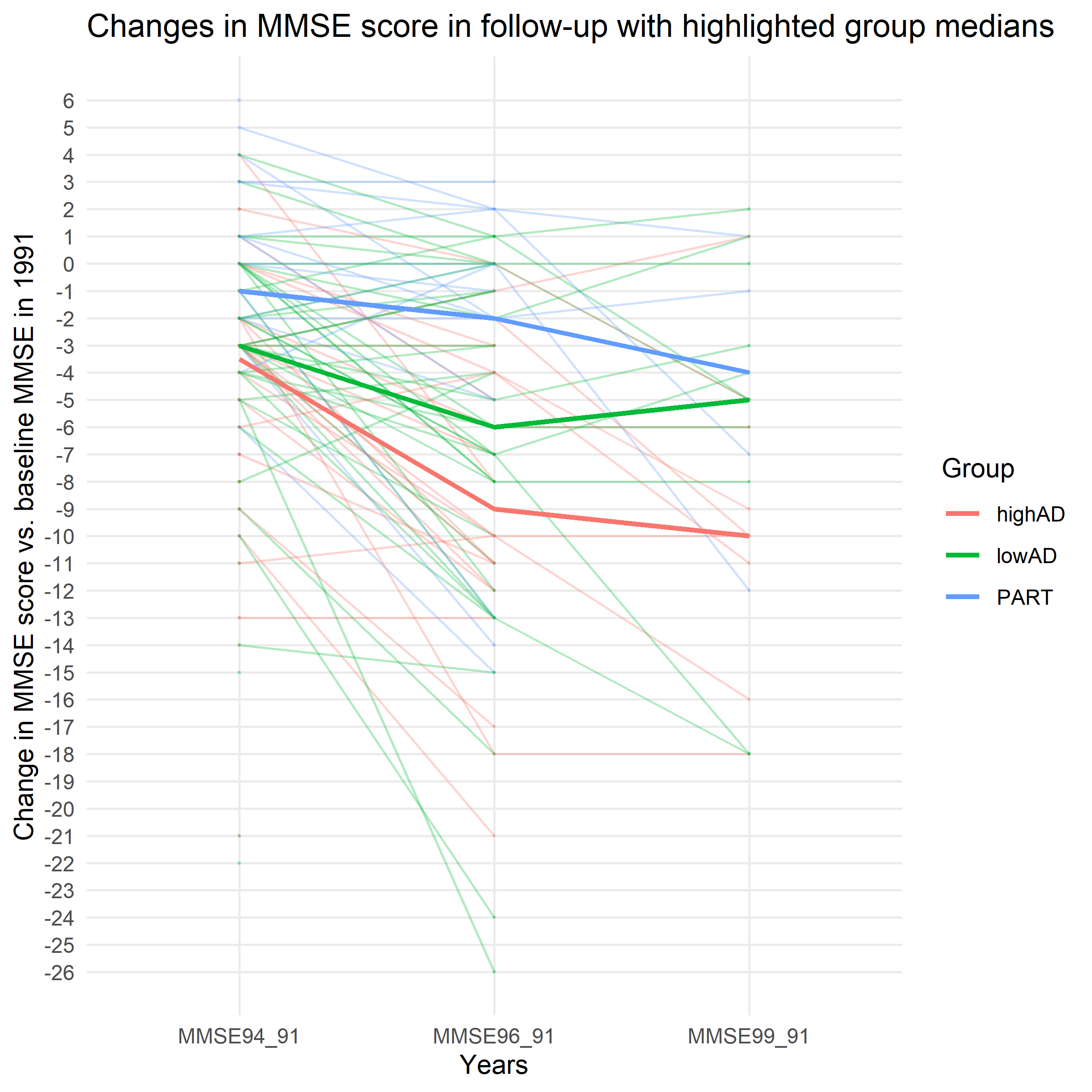


Supplementary Figure 2. Standardised AD genetic risk scores in the PART, low AD and high AD groups.


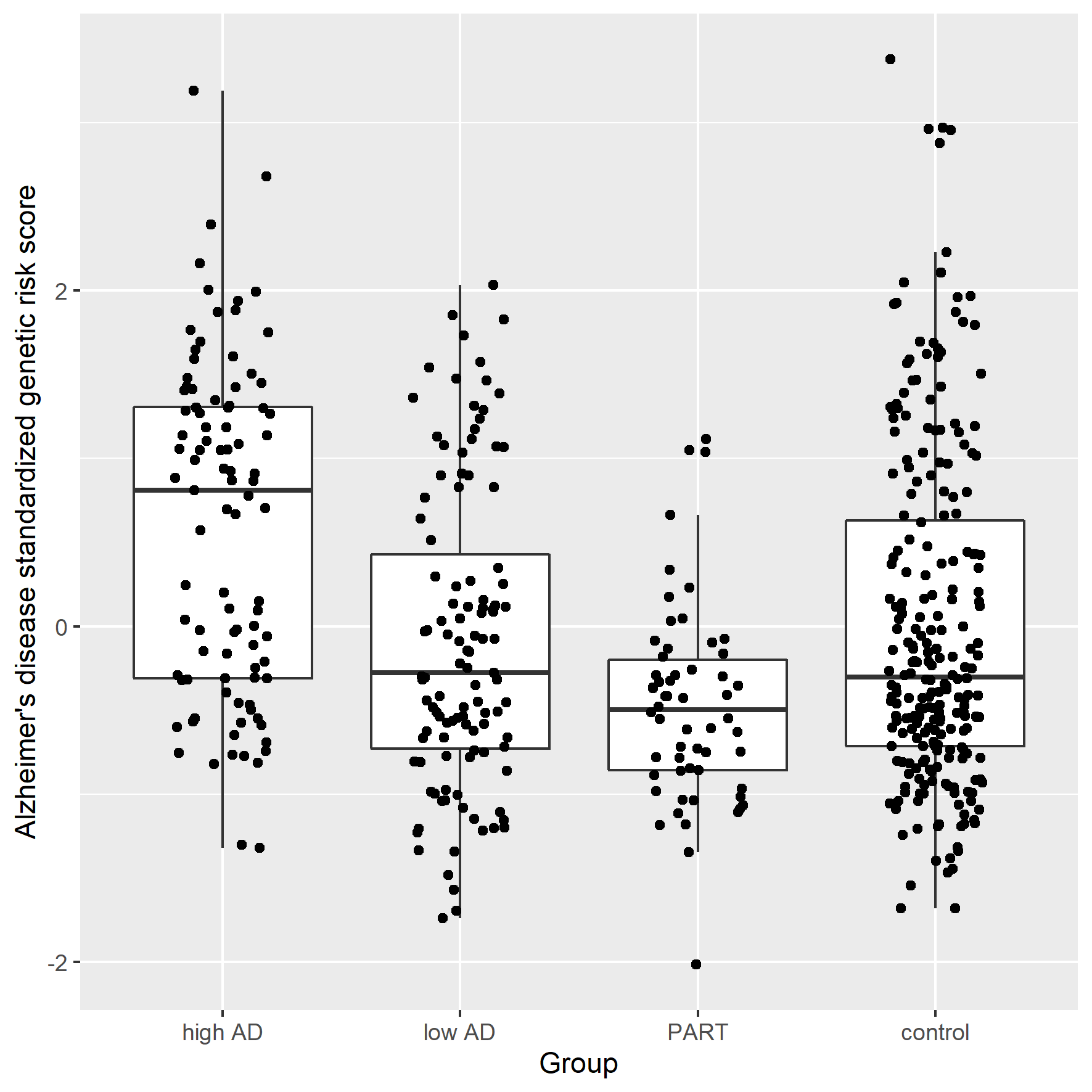


Supplementary Figure 3. Standardised AD genetic risk scores without *APOE* in the PART, low AD and high AD groups.


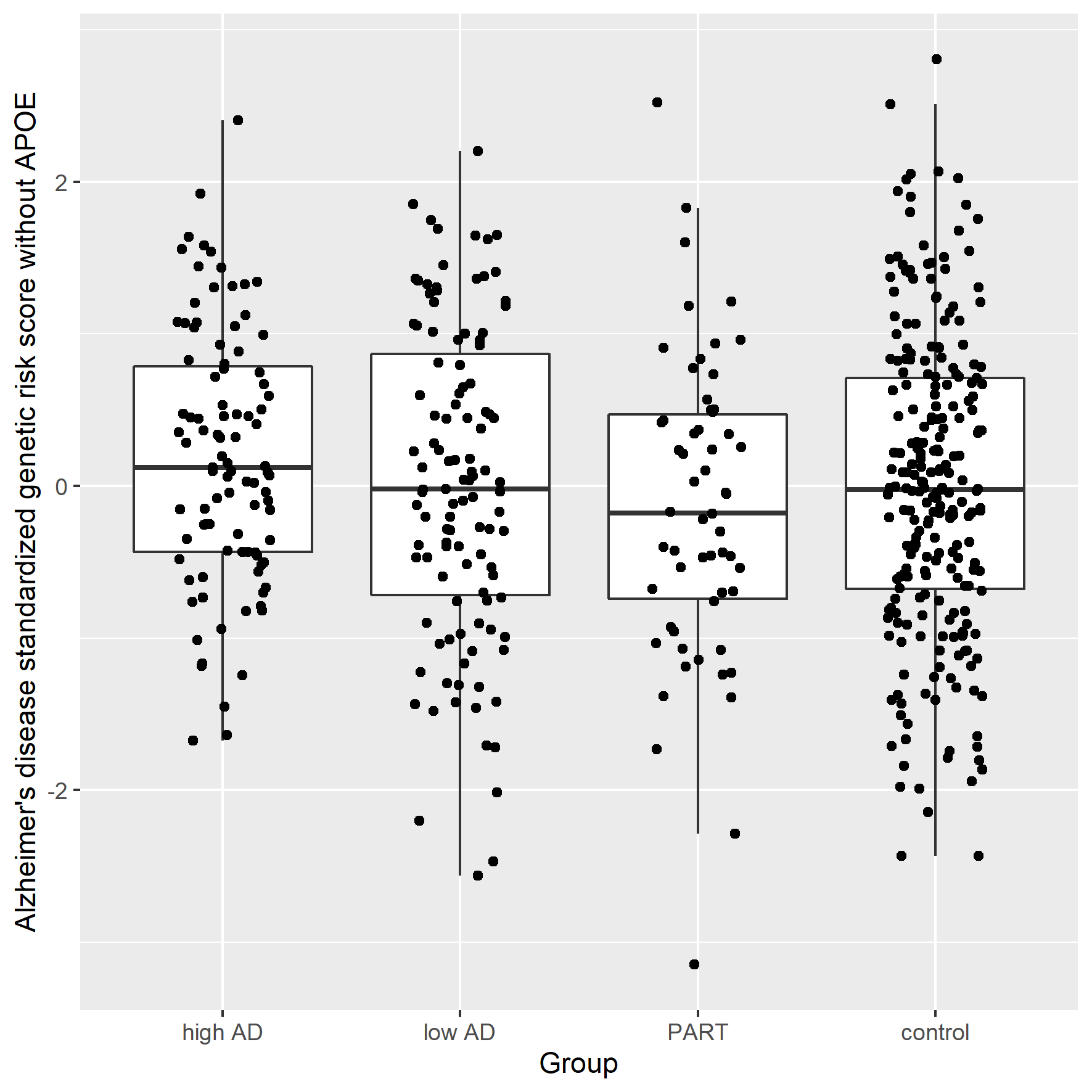


References

Laaksovirta H, Peuralinna T, Schymick JC, Scholz SW, Lai SL, Myllykangas L et al. Chromosome 9p21 in amyotrophic lateral sclerosis in Finland: a genome-wide association study. Lancet Neurol. 2010;9(10):978-85.

Santa-Maria I, Haggiagi A, Liu X, Wasserscheid J, Nelson PT, Dewar K et al. The MAPT H1 haplotype is associated with tangle-predominant dementia. Acta Neuropathol. 2012;124(5):693-704.

Kunkle BW, Grenier-Boley B, Sims R, Bis JC, Damotte V, Naj AC et al. Genetic meta-analysis of diagnosed Alzheimer's disease identifies new risk loci and implicates Aβ, tau, immunity and lipid processing. Nat Genet. 2019;51(3):414-430.

Corder EH, Saunders AM, Strittmatter WJ, Schmechel DE, Gaskell PC, Small GW et al. Gene dose of apolipoprotein E type 4 allele and the risk of Alzheimer's disease in late onset families. Science. 1993;261(5123):921-3.

Tanskanen M, Mäkelä M, Notkola IL, Myllykangas L, Rastas S, Oinas M, Lindsberg PJ, Polvikoski T, Tienari PJ, Paetau A. Population-based analysis of pathological correlates of dementia in the oldest old. Ann Clin Transl Neurol. 2017;4(3):154-65.
